# Insights Into *RP2*‐Associated Retinopathy: From Molecular Basis to Clinical Translation

**DOI:** 10.1155/humu/1839164

**Published:** 2026-10-17

**Authors:** Nina Bögershausen, Patricia Döring, Maximilian Gerhardt, Alisa Fedorenko, Carolin Strohmeyer, Rozhin Bayati, Yun Li, Gökhan Yigit, Emrah Kaygusuz, Oscar Gutiérrez-Gutiérrez, Marianne Gaubert, Behnam Javanmardi, Karin Boss, Antonia Kowallick, Peter Krawitz, Stylianos Michalakis, Marius Ueffing, Wolfram-Hubertus Zimmermann, Hans Hoerauf, Hildegard Büning, Nicolas Feltgen, Lukas Cyganek, Bernd Wollnik

**Affiliations:** ^1^ Institute of Human Genetics University Medical Center Göttingen Göttingen Germany umg.eu; ^2^ Collaborative Research Center CRC 1690 “Disease Mechanisms and Functional Restoration of Sensory and Motor Systems” University of Göttingen Göttingen Germany uni-goettingen.de; ^3^ Department of Ophthalmology University Medical Center Göttingen Göttingen Germany umg.eu; ^4^ Department of Ophthalmology LMU University Hospital LMU Medizin LMU Munich Germany uni-muenchen.de; ^5^ Stem Cell Unit Clinic for Cardiology and Pneumology University Medical Center Göttingen Göttingen Germany umg.eu; ^6^ Department of Molecular Biology and Genetics Faculty of Science Bilecik Şeyh Edebali University Bilecik Türkiye bilecik.edu.tr; ^7^ German Center for Child and Adolescent Health (DZKJ) Partner Site Göttingen Göttingen Germany; ^8^ Institute for Genomic Statistics and Bioinformatics University Hospital Bonn Bonn Germany uni-bonn.de; ^9^ Munich Center for Cell & Gene Therapy LMU University Hospital LMU Medizin LMU Munich Munich Germany uni-muenchen.de; ^10^ Institute for Ophthalmic Research Center for Ophthalmology University Hospital Tübingen Tübingen Germany uni-tuebingen.de; ^11^ Institute of Pharmacology and Toxicology University Medical Center Göttingen Göttingen Germany umg.eu; ^12^ Cluster of Excellence “Multiscale Bioimaging: From Molecular Machines to Networks of Excitable Cells” (MBExC) University of Göttingen Göttingen Germany uni-goettingen.de; ^13^ DZHK (German Centre for Cardiovascular Research) Partner Site Lower Saxony Göttingen Germany dzhk.de; ^14^ Translational Neuroinflammation and Automated Microscopy Fraunhofer Institute for Translational Medicine and Pharmacology ITMP Göttingen Germany; ^15^ Else Kröner Fresenius Center for Optogenetic Therapies (EKFZ) University Medical Center Göttingen Göttingen Germany umg.eu; ^16^ Laboratory for Experimental Gene and Cell Therapy Institute of Experimental Hematology Hannover Medical School Hannover Germany mh-hannover.de; ^17^ Eye Hospital University Hospital Basel Basel Switzerland unispital-basel.ch

## Abstract

Pathogenic variants in *RP2* cause X‐linked *RP2*‐associated retinopathy, a rare inherited retinal disorder typically characterized by childhood onset and progression to severe visual impairment within the first three decades of life. Despite its clinical severity, comprehensive clinical and genetic data on *RP2*‐associated retinopathy remain limited and no therapeutic approaches have yet progressed toward clinical application. Here, we provide an overview of current molecular, clinical, and therapeutic knowledge on *RP2*‐associated retinopathy together with an analysis of the spectrum and distribution of clinically classified, ClinVar‐curated variants. Analysis of 325 ACMG/AMP Class 3–5 variants revealed that among pathogenic and likely pathogenic variants, frameshift and nonsense variants predominated, supporting loss of RP2 function as the major disease mechanism as previously described. In contrast, variants of uncertain significance were largely missense variants, underscoring the particular challenge of missense variant interpretation in RP2. Exon distribution of *RP2* variants did not deviate from expectation for Class 3 variants, or for Class 4 and 5 variants in exons 2–4. However, we observed a significant enrichment of Class 4/5 variants in exon 1, driven mainly by initiator codon variants, and a depletion of Class 4/5 variants in exon 5. By integrating published knowledge on molecular mechanisms, genetic variants, clinical progression, and therapeutic development, with a detailed analysis of ClinVar‐annotated *RP2* variants, this narrative review outlines the current understanding of *RP2*‐associated retinopathy and identifies key gaps that will need to be addressed for therapeutic development and clinical translation.

**Trial Registration**: ClinicalTrials.gov: NCT06982417

## 1. Introduction

Retinitis pigmentosa (RP) represents a diverse group of inherited retinal dystrophies (IRD) characterized by progressive photoreceptor degeneration leading to visual impairment and often blindness [1]. Affecting approximately 1:3500 to 1:4000 individuals worldwide, RP exhibits significant genetic heterogeneity, and an ever‐growing number of genes have been implicated in its pathogenesis [2, 3]. Autosomal‐dominant, autosomal‐recessive, and X‐linked inheritance are the most common inheritance patterns [1]. Although extremely rare, mitochondrial inheritance has been observed in RP and is usually linked to syndromic presentations [4]. X‐linked RP (XLRP) accounts for approximately 10% to 15% of all RP cases [1, 5, 6]. Within this subgroup, pathogenic variants in the retinitis pigmentosa 2 (*RP2*) gene are responsible for approximately 10% to 15% of XLRP cases [7, 8]. Combining these estimates, *RP2*‐associated retinopathy accounts for approximately 1% to 2.25% of all RP cases. Applying these proportions to the estimated RP prevalence of 1:3500–1:4000 gives an estimated prevalence of approximately 1:156,000 to 1:400,000 for *RP2-*associated retinopathy, thereby clearly meeting the criteria for an ultrarare disorder, a term commonly applied to conditions with a prevalence of ≤ 1 in 50,000 [9, 10]. Given this low incidence, only a small number of *RP2-*associated retinopathy patients are seen at individual ophthalmology centers and referred for molecular genetic testing in specialized human genetics facilities. This scarcity of clinical cases limits specialist expertise across all levels of care and represents a substantial challenge for the development of targeted treatment strategies.

Preclinical studies in knockout mice and human‐derived retinal organoids have demonstrated that gene addition therapy (often also referred to as gene replacement, gene augmentation, or gene supplementation) could be a promising option to treat *RP2*‐associated retinopathy [11, 12]. However, translational efforts to date have focused mainly on the more prevalent *RPGR*‐associated XLRP, leaving *RP2-*associated retinopathy comparatively underexplored despite its severity and urgent therapeutic need.

Here, we provide an up‐to‐date narrative review of *RP2*‐associated retinopathy, encompassing clinical characteristics, genetic basis, protein function, and emerging therapeutic strategies. By combining an overview of the available literature with an analysis of clinically interpreted *RP2* variants, this work is aimed at refining the current understanding and support future translational research efforts in *RP2-*associated retinopathy.

## 2. Methods

### 2.1. Literature Search and Narrative Review

This article was designed as a narrative literature review complemented by a quantitative analysis of clinically interpreted *RP2* variants in ClinVar [13]. Given the rarity of *RP2-*associated retinopathy and the heterogeneous nature of the available literature, a narrative approach was considered appropriate. The available evidence comprises retrospective cohort studies, family studies, case reports, molecular and functional studies, experimental models, and emerging therapeutic studies, which differ substantially in study design, cohort size, and depth of clinical and genetic characterization.

Relevant literature was identified through targeted PubMed/MEDLINE searches addressing six topic areas: (1) male clinical phenotype and natural history, (2) phenotype of female carriers, (3) genetics and genotype–phenotype correlations, (4) molecular and cellular function, (5) experimental models, and (6) therapeutic approaches. Search terms are provided in Table S1. The final search was performed on August 18, 2026, using PubMed/MEDLINE as the only bibliographic source.

Because of the narrative nature of this review, the final article selection was curated manually to prioritize papers with the highest relevance to the topic. Only peer‐reviewed publications written in English were included. Publications were included when they contributed directly to the clinical, genetic, molecular, experimental, or therapeutic aspects of *RP2*‐associated retinopathy. Because of the rarity of the condition, cohort studies, family series, and individual case reports were considered alongside molecular, experimental, and therapeutic studies. General retinal‐dystrophy literature was used selectively to provide relevant context. Reference lists of relevant publications were also screened to identify additional relevant publications. Publications in which *RP2* was mentioned only incidentally or that did not contribute information relevant to the scope of the review were excluded.

Clinical and genetic studies were categorized according to study type, including gene identification, mutation screening, family studies, case reports, genotype–phenotype studies, natural history studies, and meta‐analyses. Relevant study characteristics are summarized in Table S2. Detailed clinical characteristics are presented in Tables S3 and S4.

### 2.2. ClinVar Data Extraction

ClinVar (https://www.ncbi.nlm.nih.gov/clinvar/) was searched for the gene name “*RP2*” [13]. *RP2* variants were selected based on the annotated clinical significance according to the ACMG/AMP variant classification guidelines [14]. The ACMG/AMP criteria provide a standardized framework for classifying genetic variants based on the combined evidence for their pathogenicity, including population, computational, functional, clinical, and segregation data, defining a five‐tier system as benign (Class 1), likely benign (Class 2), variant of uncertain significance (Class 3), likely pathogenic (Class 4), or pathogenic (Class 5) [14]. Variants classified as pathogenic (Class 5), likely pathogenic (Class 4), or variants of uncertain significance (Class 3) were included, whereas variants classified as benign (Class 1) or likely benign (Class 2) were excluded. Variants with conflicting interpretations of pathogenicity were excluded. Review status was recorded in Table S5 but was not used as an additional exclusion criterion.

ClinVar VCV (Variation ClinVar) records were retrieved in three groups according to variant size and type. First, short variants (< 50 bp) were retrieved. Second, structural variants > 50 bp affecting a single gene (< 1 kb) were retrieved. Third, larger structural variants (> 50 bp) were retrieved separately and manually screened for the phenotype annotation “retinal dystrophy.” One deletion was retained from this group based on its relevance to *RP2*‐associated retinal disease. Large deletions encompassing larger parts of chromosome X and associated with multiple syndromic phenotypes were excluded to focus the analysis on *RP2*‐specific disease mechanisms.

The retrieved records were manually reviewed for duplicate entries at the nucleotide level. Variants representing the same amino acid change but arising from different nucleotide changes were retained as distinct variants, as the analysis was performed at the DNA sequence level. Each distinct *RP2* variant was counted once, irrespective of the number of ClinVar submissions or published reports associated with that variant, to avoid overrepresentation of recurrently reported variants. The final dataset comprised 325 distinct *RP2* variants. The complete dataset underlying the analysis, including ClinVar identifiers, variant nomenclature, transcript, molecular consequence, clinical significance, review status, phenotype annotation, and other relevant metadata, is provided in Table S5. The ClinVar database was last accessed on August 19, 2026, at 11:53 a.m. (UTC+2).

Variants were categorized according to variant type and their location in specific exons and neighboring intronic regions. Initiator codon variants (variants affecting the first ATG triplet) were analyzed as a separate functional category, although they may be annotated as missense or frameshift variants in ClinVar to reflect their distinct molecular consequences.

Because the primary analysis counted each distinct nucleotide‐level variant only once, information on repeated clinical submissions was not initially captured. We therefore additionally identified variants with multiple ClinVar submissions as a descriptive observation. The same filtering criteria described above were applied, followed by an additional filter for variants with “multiple submitters.” This yielded 50 relevant variants, all of which were short variants (< 50 bp). One additional structural variant (> 50 bp) identified by the multiple‐submitter filter was excluded because the deletion encompassed multiple genes and had no phenotype annotation. The 50 retained variants (Table S5) were manually reviewed in ClinVar to document the number of available submissions, submitter information, and associated phenotype. Submitter information was reviewed to determine whether multiple submissions originated from different laboratories and therefore might represent independent clinical observations.

### 2.3. Gene and Protein Structure

Exon sizes and gene structure were extracted from Ensembl (http://www.ensembl.org/index.html) and based on canonical transcript ENST00000218340.4, corresponding to RefSeq transcript NM_006915.3. Protein structure was based on Uniprot ID O75695 (https://www.uniprot.org/). RP2 3D‐structure AF‐O75695‐F1‐v6 was retrieved from AlphaFold (https://alphafold.com/entry/AF-O75695-F1; last accessed on August 26, 2026) [15].

### 2.4. Statistical Analysis of Variant Distribution Across Exons

To assess whether *RP2* variants are distributed proportionally to exon size, we compared observed variant counts per exon with expected counts based on exon length. Expected counts were calculated as $E_{i}=N\left(\;L_{i}/\sum_{j=1}^{5}L_{j}\right)$, where *N* is total number of variants and *L*_*i*_  is the coding sequence length of exon *i*, based on ENST00000218340.4. Analyses were performed separately for deduplicated ACMG/AMP Class 4/5 variants and ACMG/AMP Class 3 variants. One hundred and seventy five small variants were included in the statistical analysis of exon distribution for the Classes 4 and 5, while the whole gene deletion was excluded. In Class 3, 133 point mutations localized within exons or at exon/intron boundaries were included, and untranslated region (UTR) variants were excluded. For the analysis of variant type composition, all 325 variants were considered. The complete variant lists are provided in Table S5.

Observed and expected counts were compared using a Pearsons′s chi‐square goodness‐of‐fit test with four degrees of freedom. As a robustness analysis, we also calculated the likelihood ratio G‐test against the same reference distribution. Before interpreting the chi‐square results, Cochran′s criterion was assessed: No expected cell count was below 1, and no more than 20% of cells had expected counts below 5. Because exon 5 contained a very small observed count in the Class 4/5 analysis, the corresponding global result was additionally evaluated using a Monte Carlo goodness‐of‐fit test based on 200,000 simulated tables, which does not rely on the large‐sample chi‐square approximation [16, 17].

To identify which exons contributed most to any overall deviation, standardized Pearson residuals were calculated for each exon, and each exon′s contribution to global chi‐square statistic was determined. Residuals were converted to two‐tailed *p* values, followed by Bonferroni correction across the five exons; significance was defined as exons with adjusted *p* values below 0.05.

To assess whether the results were driven by any particular variant type, sensitivity analyses were performed by iteratively excluding each variant type in turn and recalculating the expected counts, test statistics, and exon‐specific residuals for the remaining variants. Near‐exonic intronic variants were grouped with the adjacent exon to facilitate exon‐based statistical analysis. All calculations were performed in Python using the SciPy statistical library (Data S1).

### 2.5. Informed Consent

Written informed consent for publication of the clinical images in Figure 1 was obtained from the patient′s legal guardians.

**Figure 1:**
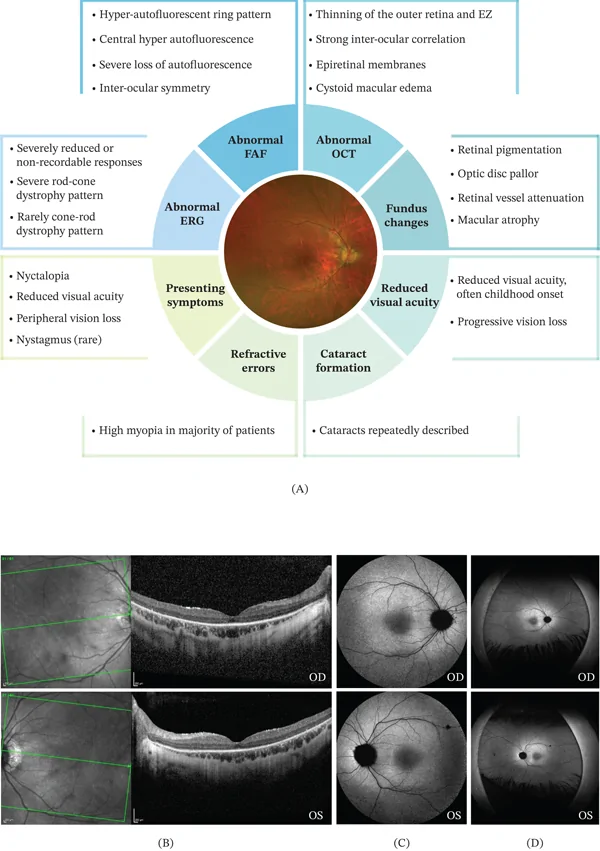
Clinical hallmarks of *RP2-*associated retinopathy. (A) Overview of symptoms and clinical features of *RP2-*associated retinopathy. Inner circle: Optos color fundus image (OD) of a 17‐year‐old male individual with *RP2-*associated retinopathy. Note generalized dystrophic fundus appearance. (B) Optical coherence tomography (OCT) images showing symmetrical bilateral photoreceptor atrophy with marked thinning of the outer nuclear layer and ellipsoid zone. Note the small islet of relatively preserved retinal structure in the foveola centralis. (C) 55° fundus autofluorescence (FAF) images showing symmetrical bilateral hyperautofluorescence at the posterior pole, pronounced in areas with higher rod photoreceptor density. Reduced autofluorescence is observed in the fovea‐surrounding region, presenting as an enlarged hypoautofluorescent area. (D) Ultrawidefield fundus autofluorescence (FAF) images acquired using an Optos system. Note that the central hyperautofluorescence pattern becomes more evident in wide‐angle FAF. Eye laterality (OD/OS) is indicated in each panel. Images in Panels B–D are representative of a single individual. Abbreviations: ERG, electroretinography, EZ, ellipsoid zone, FAF, fundus autofluorescence, OCT, optical coherence tomography, OD, oculus dexter, OS, oculus sinister.

## 3. Results

### 3.1. Natural History

#### 3.1.1. Introduction and Terminology

*RP2*‐associated retinopathy is an early‐onset form of XLRP, characterized by a rapid decline in visual function and early macular involvement in most cases, although a certain degree of variability in onset, progression, and retinal phenotype has been described. Its retinal phenotype has been mainly described as RP, as well as rod‐cone or cone‐rod dystrophy, Leber congenital amaurosis, and other, rarer phenotypic variants, such as macular or peripapillary phenotypes [18–22]. RP describes a predominantly rod‐driven retinal degeneration, typically presenting with night blindness and progressive peripheral visual‐field loss, followed by increasing involvement of central vision. Rod‐cone dystrophy similarly refers to predominant rod dysfunction with subsequent cone involvement and is often used interchangeably with RP in the context of *RP2* variants. Cone‐rod dystrophy describes the opposite pattern, with cone dysfunction and central visual impairment preceding or exceeding rod dysfunction. Leber congenital amaurosis represents a severe, early‐onset retinal dystrophy characterized by markedly impaired vision from infancy or early childhood. Other, rarer phenotypic presentations have also been reported. In this review, we use the term *RP2*‐associated retinopathy as an umbrella term to capture this phenotypic spectrum.

#### 3.1.2. Age of Onset

The typical age of onset in affected males is early childhood, although later presentation can occur. In the largest genetically characterized cohort, Georgiou et al. [19] identified 54 molecularly confirmed patients from 38 pedigrees, of whom 53 had an RP phenotype, whereas one individual had a presentation consistent with cone‐rod dystrophy. Among the 48 patients with available age‐of‐onset data, 44 (91.7%) had childhood‐onset disease, with a mean age of onset of 7.6 years (SD 4.1), whereas 4/48 (8.3%) had adult‐onset disease, with a mean age of onset of 32.5 years (SD 16.2). Cheloni et al. [18] reported a median age at first symptoms of 7 years (IQR 2.25–12) among 14 patients with available data. In the Japanese cohort of Fujinami et al. [23], age at onset was available for 3/4 affected males and ranged from 3 to 28 years (mean 11.3 years). A review of previously reported Japanese cases identified age‐of‐onset data for eight affected males, with a mean onset of 5.75 years (range: 3–11 years) [23]. A case report by Choi [24] described symptom onset as early as age four in two male siblings.

#### 3.1.3. Presenting Symptoms

Nyctalopia (night blindness) is consistently the most frequently reported initial symptom. In Georgiou et al. [19], presenting symptoms were documented for 48 patients: 33/48 (68.8%) reported night blindness, 8/48 (16.7%) decreased central vision, 4/48 (8.3%) both night blindness and decreased central vision, and 2/48 (4.2%) decreased central and peripheral vision. Only one patient presented with nystagmus. Similarly, nyctalopia was reported in 16/23 patients (69.6%) in Cheloni et al., for whom this information was available. Three of 23 (13.0%) reported reduced vision, 2/23 (8.7%) both reduced vision and nyctalopia, and one patient each presented with nystagmus or was asymptomatic [18]. Ponjavic et al. [25] reported night blindness as the first subjective symptom in all three affected males in one *RP2* family. Fujinami et al. [23] reported night blindness as the presenting symptom in two of four male patients, whereas photophobia and reduced visual acuity were reported as the initial symptom in one patient each.

#### 3.1.4. Best‐Corrected Visual Acuity (BCVA)

BCVA is substantially affected in *RP2-*associated retinopathy, although the available data differ between studies. In Georgiou et al., the mean BCVA at baseline was approximately 0.91 logMAR, declining to 1.16–1.17 logMAR during a mean follow‐up of 7.3 years. According to WHO criteria, 18/53 patients (34%) had low vision and 11/53 (21%) were blind [19]. Cheloni et al. reported baseline BCVA for all 47 affected males, with counting‐fingers vision or worse in 10/47 (21%). Longitudinal analysis was possible in 41/47 patients, who had a median follow‐up of 8.0 years (IQR 3.2–14.5; median number of visits 2, IQR 2–5). Median BCVA declined from 0.66 logMAR (IQR 0.35–1.4) at baseline to 1.3 logMAR (IQR 0.6–1.4) at the last visit, corresponding to an estimated rate of visual loss of 46%–49% per decade [18]. In Jayasundera et al. [20] BCVA data were available for 11 patients, of whom nine had BCVA worse than 20/50 before the age of 12 years. Sharon et al. [26] reported visual acuity in 16 patients with *RP2* variants (mean age 27.0 ± 4.3 years). The age‐adjusted geometric mean BCVA was 20/210, and visual acuity declined with increasing age.

#### 3.1.5. Fundus Imaging

Fundus abnormalities were consistently reported across studies. Cheloni et al. obtained gradable fundus photos for 37 patients and documented classic signs of RP, including intraretinal pigmentation in 33/37 patients (89.2%), attenuation of retinal vessels in 35/37 (94.6%), and optic disc pallor in 10/37 (27.0%), as assessed by wide‐field pseudocolor fundus imaging. They also noted macular changes in 22/37 patients (58.5%). Patients with visible macular abnormalities were older (median 32 years vs. 11 years) and had poorer BCVA (median 1.4 vs. 0.3 logMAR) than those without macular changes [18].

Jayasundera et al. reported that 11/12 (92%) of males had macular granularity, atrophy, or bull′s‐eye appearance. Macular changes were seen on fundus images before the age of 12 in 10 out of 11 patients (91%). Tapetal or golden macular sheens were not observed, and the authors noted this to be a distinctive feature from *RPGR*‐associated XLRP, where these changes are seen frequently. Moreover, they described two cases with a choroideremia‐like fundus characterized by midperipheral choriocapillaris atrophy in the absence of *CHM* variants, suggesting that a choroideremia‐like appearance could be indicative of *RP2-*associated retinopathy [20].

Ponjavic et al. described typical RP fundus changes in three affected males, including bone‐spicule pigmentation in all four peripheral quadrants, attenuated retinal vessels, and optic disc pallor. The fundus degeneration progressed with age, whereas the macula remained normal in all three affected males [25], indicating a certain degree of variability regarding macular abnormalities.

Fujinami et al. described variable aspects in fundus appearance among four affected males. Patient 1 had extensive retinal atrophy with a relatively preserved foveal appearance and attenuated retinal vessels. Patient 2 showed extensive atrophy with peripheral bone‐spicule pigmentation, patchy macular pigmentation, vessel attenuation, and optic disc pallor. Patient 3 had predominantly peripheral atrophy with a relatively preserved fovea, accompanied by vessel attenuation and optic disc pallor. In contrast, Patient 4 had atrophic changes predominantly at the posterior pole with macular pigmentation, whereas the peripheral fundus remained relatively preserved [23].

#### 3.1.6. Optical Coherence Tomography (OCT)

Structural changes observed on OCT are striking and progressive. Georgiou et al., as well as Cheloni et al. reported rapid degeneration of the outer nuclear layer (ONL) and ellipsoid zone (EZ). Cheloni et al. found that at baseline 23/35 patients (65.7%) with gradable OCT showed severe disruption of the EZ and outer retinal atrophy, whereas 12/35 (34.3%) retained a measurable EZ. Patients with a measurable EZ were significantly younger, with a median age of 14.5 years (IQR: 12–21.8), compared with 35 years (IQR: 26–48) among those with severe EZ disruption. Among the 12 patients with a measurable EZ, the median EZ width was 564.5 *μ*m (IQR: 485–903) [18]. Longitudinal OCT analysis was possible in 27 patients with two or more OCT examinations over a period of 3–7 years (median 6 years), showing a significant decline in central retinal thickness (CRT) with age. The median CRT was 118 *μ*m at baseline (IQR: 94 to 145), dropping to a median of 96 *μ*m (IQR: 83 to 129.5) at follow‐up, corresponding to an estimated loss of 12.6% per decade. In this patient group, the median photoreceptor and retinal pigment epithelium complex (PR + RPE) thickness was 41.5 *μ*m (IQR: 25 to 70.25) at baseline, and 25 *μ*m (IQR: 18 to 38) at follow‐up. Both measurements showed a strong interocular correlation and a significant linear effect of age, with a 33.9% decline per decade [18]. In Georgiou et al., OCT imaging was available for 46/54 patients, including 42 with childhood‐onset disease and four with adult‐onset disease. Among the 42 childhood‐onset patients, 23 had already lost the EZ and showed complete loss of measurable ONL thickness, whereas 19/42 retained both an identifiable EZ and residual ONL. The latter group had a mean age of 13.5 ± 5.7 years (range: 5.3–25.9), compared with 36.4 ± 16.0 years (range: 17.9–69.2) among those with complete EZ and ONL loss. Longitudinal OCT data were available for 16/19 patients with preserved EZ and ONL over a mean follow‐up of 5.3 years, during which EZ width decreased by an average of 219 *μ*m/year and ONL thickness by 4.93 *μ*m/year. No childhood‐onset patient retained a detectable EZ beyond 26 years of age. Among the four adult‐onset patients, three had longitudinal imaging; one patient with onset at 17 years subsequently lost the EZ completely over 7.4 years, whereas the two patients with later onset showed stable imaging over 1.4 and 2.9 years, respectively [19]. Fujinami et al. reported photoreceptor‐layer loss extending throughout the retina in 3/4 patients, whereas a relative preservation of the foveal photoreceptor layers was seen in one patient. In two affected brothers described by Choi et al., OCT similarly demonstrated diffuse retinal thinning and EZ disruption (2/2, ages 14 and 16 years). Additional features observed on OCT imaging were epiretinal membranes (17/35 patients, 48.5%) and cystoid macular edema (1/35 patients, 2.8%) in the Cheloni cohort. Representative OCT images of an individual with *RP2-*associated retinopathy are shown in Figure 1B.

#### 3.1.7. Fundus Autofluorescence (FAF)

FAF imaging showed a variable phenotype. Cheloni et al. obtained FAF images for 43/47 patients, of which 38 were gradable. They found severe loss of autofluorescence in 21/38 patients (55.3%). A hyperautofluorescent ring pattern was observed in 9/38 patients (23.8%); these patients were younger than those with central hyperautofluorescence or severe loss of autofluorescence (median age 15 vs. 26 years), and seven of these patients (77.8%) still had a measurable EZ on OCT. Central hyperautofluroescence was observed in 6/38 patients/15.8%). A minority of patients (2/38, 5.3%) presented with granular mottled patches of hypoautofluorescence limited to the periphery, with seemingly intact FAF at the posterior pole [18].

In the study of Georgiou et al., FAF imaging was available for 46/54 patients. Atrophy was the most common pattern, observed in 18/46 (39.1%) patients, whereas 11/46 (23.9%) had normal FAF. Additional patterns reported were a paracentral macular ring of increased signal in 2/46 patients, a macular ring of increased signal and midperipheral patchy changes in 6/46 (13.0%), a patchy macular signal and midperipheral changes in 4/46 (8.7%), or a normal macular signal with patchy midperipheral changes in 4/46 (8.7%). One patient had a patchy macular signal with normal periphery. Three of the 18 patients with advanced atrophy showed a choroideremia‐like pattern. Overall, 31/46 patients (67.4%) had visible macular FAF abnormalities at baseline. Follow‐up FAF was available for 36 patients over a mean of 6.0 years (± 4.3, 0.6–17.6 years), of these 9/9 patients with normal FAF at baseline developed abnormalities on follow‐up. A high degree of interocular symmetry was reported for FAF findings [19]. Representative FAF images of an individual with *RP2-*associated retinopathy are shown in Figure 1C.

#### 3.1.8. Electrophysiology

*RP2-*associated retinopathy shows marked retinal dysfunction, with electrophysiological findings generally consistent with a severe rod‐cone dystrophy and substantial macular dysfunction. In the cohort of Georgiou et al., full‐field ERG and pattern ERG (PERG) were performed in 22 patients. Seventeen of these 22 patients showed an ERG phenotype consistent with rod‐cone dystrophy. PERG P50, used as an objective measure of macular function, was undetectable in all but 2/22 patients, indicating severe macular dysfunction in most patients undergoing electrophysiological assessment [19]. The patients undergoing ERG testing were predominantly young (5–21 years of age). Serial data, which were available for one child, showed a progressive reduction in PERG P50 between 7 and 12 years of age, accompanied by marked worsening of the dark‐adapted 10 ERG between 10 and 12 years [19].

The findings reported by Jayasundera et al. are consistent with this severe functional phenotype: Standardized ERG was available for 10 patients, of whom nine (90%) showed severe rod‐cone dysfunction, whereas the remaining patient had a cone‐rod pattern [20].

Similarly, Choi et al. [24] demonstrated nearly flat ERG responses in two affected brothers at ages 16 and 14 years, respectively, indicating profound cone and rod dysfunction. Sharon et al. reported reduced 30‐Hz full‐field ERG amplitudes in 13/16 patients with *RP2* variants (mean age of the *RP2* cohort, 27.0 ± 4.3 years). The age‐ and refractive‐error‐adjusted geometric mean amplitude was 1.93 mV, compared with a normal reference of ≥ 50 mV. ERG amplitude declined with increasing age [26]. Electrophysiology findings were only available in a minority of patients in the Cheloni et al. study and therefore not analyzed in detail. However, the authors mention a severe loss of ERG response at baseline [18].

#### 3.1.9. Visual Fields

Visual‐field abnormalities were documented in several cohorts and family reports, although the level of reporting differs greatly between studies. The largest available clinical cohort studies by Cheloni et al. and Georgiou et al. do not report systematically on visual‐field tests. Cheloni et al. stated that visual‐field examinations were available in only a minority of patients and generally showed severe loss at baseline [18].

Jayasundera et al. provided visual‐field data for 10 individuals and identified central scotomas in 5/10 affected males (50%) who underwent Goldmann visual‐field (GVF) testing, including 3/8 (38%) of those younger than 12 years. Among eight boys younger than 12 years 6/8 (75%) showed I4e field constriction, with median field sizes of 25° in both eyes, whereas IV4e fields showed only mild constriction (median 55° in both eyes). When the authors considered measurements from patients up to the age of 16 years, the median I4e field size was 12.5° OD and 17.5° OS, whereas the median IV4e field size was 50° in both eyes.

Ponjavic et al. described progressive visual‐field constriction in all three affected males of a Swedish family, with substantial differences in the pattern of field loss at initial examination. The youngest patient (IV‐3, 17 years) had constricted peripheral fields in both eyes, with preserved temporal islands in the inferior temporal quadrants. His older brothers (30 and 22 years at last examination) had only central visual fields, measuring approximately 5°–10° with the V4e target in Patient IV‐1 and 10°–20° in Patient IV‐2. [25].

Kurata et al. reported quantitative visual‐field measurements in six affected males. The median V4e field area was 10,814 mm^2^ (range: 5528–20,048), and the median I‐4e field area was 3 mm^2^ (range: 0–52). V‐4e field area declined significantly with age (*r* = −0.79, *p* < 0.001) [27].

Sharon et al. evaluated visual‐field area in 15 affected males (mean age 27.0 ± 4.3 years) using GFV. The age‐adjusted geometric mean visual‐field area was 3640 deg^2^ (square degrees), compared with a normal reference of ≥ 11,399 deg^2^, indicating substantial visual‐field loss [26].

Fujinami et al. performed GVF in four affected males from their study. Two patients had peripheral field loss with a central scotoma, one had an entire visual‐field defect, and one had a large central scotoma with preserved peripheral vision [23], indicating interindividual variability.

#### 3.1.10. Cataracts

Cataract formation, most commonly of the posterior subcapsular type, is a frequent secondary finding in RP and tends to occur at a significantly younger age than in the general population [28]. Cataracts also occur in *RP2-*associated retinopathy, but detailed data on cataract morphology and age of onset remain sparse. Cheloni et al. [18] reported cataracts in 8/22 patients (36.4%) with available baseline right‐eye lens‐status data, whereas 5/22 (22.7%) had pseudophakia, and 9/22 (40.9%) had a clear lens. In a family described by Ponjavic et al. [25], all three affected males showed early‐onset cataract formation described as “atypical with multiple opacities,” and the youngest had suspected amblyopia of the left eye, reportedly secondary to early cataract. The patients were 17, 22, and 30 years old at last examination, respectively [25]. Cataracts were also reported in two affected males (ages 44 and 48 years) in a family described by Mashima et al. (2001), where the opacities were characterized as “dense” [29]. Fujinami et al. found cataracts in two out of four patients, who were 35 and 47 years old, respectively, at last examination [23].

#### 3.1.11. Refractive Errors

In addition to the aforementioned symptoms, *RP2-*associated retinopathy has been reported to be characterized by high myopia. In the study by Cheloni et al., refractive measures were available for 10 patients. These patients had a median spherical equivalent of −7.7 diopters (D) (IQR: −8.3 to −6.2 D) [18]. Kurata et al. reported a median refractive error of −5.8 D (range: −10.0 to +1.00 D) in six male affected individuals. Jayasundera et al. presented refractive‐error data for 11/15 patients, ranging from plano to −14 D (mean −6.55 D). Nine of these 11 patients (82%) were classified as myopic, with a mean refractive error of −7.97 D, whereas 7/9 (78%) of the myopic patients were classified as highly myopic (> −6.00 D), with a mean refractive error of −8.91 D [20].

#### 3.1.12. Progression and Variability

Although the typical course of *RP2-*associated retinopathy is characterized by early onset and rapid progression, phenotypic variability exists. Georgiou et al. documented 4/48 individuals with adult‐onset disease, with a mean age of onset of 32.5 years (range: 17–57 years). Although two patients with later onset had stable OCT findings over 1.4 and 2.9 years, respectively, the patient with onset at 17 years progressed to complete EZ loss over 7.4 years [19]. Similarly, Cheloni et al. described a range of BCVA and imaging profiles at baseline, with some patients retaining central EZ bands and foveal function well into adulthood [18]. Phenotypic variability is also evident in electrophysiological findings. Georgiou et al. reported that full‐field ERG severity varied considerably among children and adolescents of similar age, ranging from undetectable rod and cone responses to near‐normal cone‐mediated responses, and pointed out that phenotypic variability is considerable especially in the first two decades of life [19]. However, most male patients exhibit symmetrical bilateral involvement and rapidly declining vision in the second and third decades [18, 27]. Although most patients with *RP2-*associated retinopathy show a rod‐cone type of retinal dystrophy, atypical cases with primarily macular and/or peripapillary retinal dystrophy have also been described [19–21].

### 3.2. Genotype–Phenotype Correlations in Males

Although smaller studies suggested that missense variants might be associated with a milder phenotype compared with truncating variants [20, 23], this finding could not be reproduced in larger cohorts [18–20]. Rosenberg et al. [30] reported marked interfamilial differences, with severe phenotypes associated with both the nonsense variant p.Gln26Ter and the missense variant p.Arg118His, and a milder phenotype in association with the in‐frame *Δ*Ser6 deletion. Fujinami et al. [23] reported an association between variant class and phenotype in a smaller Japanese cohort, with a statistically significant association between genotype group and phenotype severity. However, Jayasundera et al. [20] found severe clinical presentations in all nine patients with nonsense or frameshift variants and in 5/7 patients (71%) with missense variants, demonstrating that missense variants could also be associated with severe disease. Saeed et al. performed a meta‐analysis of reported cases published between 1992 and 2019. They could not reproduce previously reported genotype–phenotype correlations and attributed this to the small sample size. However, Cheloni et al. [18] reported similar findings in their 2022 cohort study, identifying 24 distinct *RP2* variants (13 novel), yet finding no pattern linking variant class to clinical outcome. In 2023, Georgiou et al. published the largest clinically characterized cohort yet and found no genotype–phenotype correlation in their cohort. They noted that null and missense variants occurred in both childhood‐ and adulthood‐onset disease; notably, two of the four patients with adulthood‐onset disease carried frameshift variants predicted to cause truncation/loss of function, whereas the other two carried a splice‐site variant and a missense substitution [19]. Thus, no clear genotype–phenotype correlations have yet been established for *RP2*‐associated retinopathy.

### 3.3. Female Carriers

#### 3.3.1. Clinical Observations

The phenotype of female carriers of causative *RP2* variants is highly variable and ranges from absence of visual complaints or structural retinal changes to clinically manifest retinal dystrophy. Georgiou et al. [31] examined 27 female carriers from 21 pedigrees. Four carriers reported visual symptoms, whereas 23/27 (85.2%) did not report visual complaints. The mean age was similar in the two groups: 42 years (range: 16–76) among carriers without complaints and 41 years (range: 30–51) among those reporting visual symptoms. Among the four symptomatic carriers, two patients had mild disease, whereas two had severe disease according to the clinical severity grading used by the authors. Using WHO visual‐impairment criteria, 24/27 carriers (88.9%) had no or mild visual impairment, 1/27 (3.7%) had moderate visual impairment, and 2/27 (7.4%) were blind [31]. Fundus examinations showed that 8/27 carriers had a normal fundus, 10/27 had a tapetal‐like reflex, and 5/27 had scattered peripheral pigmentation, indicating that subclinical abnormalities (i.e. imaging abnormalities in the absence of perceived visual complaints) are relatively common in carriers. The four symptomatic carriers had fundus findings typical of RP. Full‐field ERG was available in 11 carriers (median age 38 years, range: 21–67). Responses ranged from bilaterally normal to undetectable, with varying degrees of rod and cone dysfunction. Interocular amplitude asymmetry was frequent and reached up to 41%. PERG P50 was normal in most tested carriers but was reduced or absent in those with the most severe full‐field ERG abnormalities [31].

Kurata et al. examined five female carriers of *RP2* variants, with a median age of 58 years (range: 47–73 years). Visual‐disturbance information was available for four carriers, of whom 2/4 (50%) reported visual disturbances. Median decimal BCVA was 0.9 (range: 0.2–1.5), whereas median refractive error was −0.25 D (range: −11.0 to +1.75 D). Visual fields were relatively well preserved, with median V‐4e and I‐4e field areas of 21,253 mm^2^ (range: 18,339–22,501) and 10,113 mm^2^ (range: 8,464–13,711), respectively. Fundus appearance was graded from 0 to 3, ranging from a normal‐appearing fundus (Grade 0), through a tapetal‐like reflex or regional peripheral pigmentary changes (Grades 1–2), to diffuse pigmentary disturbance with retinal atrophy (Grade 3). Among eight examined eyes, 2/8 were Grade 0, 4/8 Grade 1, and 2/8 Grade 2; none were Grade 3 [27].

Jayasundera et al. described three female *RP2* carriers. All three had macular atrophy in one or both eyes and were myopic, with a mean refractive error of −6.23 D. Two carriers showed a phenotype similar to that of affected males, with atrophic macular changes, reduced visual acuity, and central scotomata. The third carrier had markedly asymmetric disease, with approximately 8 D of anisometropia, with the more severely affected eye being myopic [20]. Sun et al. described five female carriers of the same *RP2* variant in a Chinese pedigree, aged 25–54 years (mean, 35.5 years) at baseline. Only one carrier (III‐2, aged 32 years) had relatively normal visual function and retinal structure, whereas the other four showed retinal abnormalities. Across the 10 eyes, 8/10 had reduced BCVA and 7/10 were myopic. Fundus abnormalities included tessellation in 7/10 eyes, hyperpigmentation in 6/10, vessel attenuation in 6/10, and retinal atrophy in 2/10. ERG abnormalities were detected in 8/10 (80%) eyes. Three carriers had phenotypes approaching those of the affected males [32]. Saeed et al. performed a cross‐sectional meta‐analysis of 35 female carriers with confirmed *RP2* variants from 13 previously published studies. Several of the primary studies included in this review contributed cases to the analysis. The carriers had a mean age of 44.2 ± 17.4 years (median 46.5, range: 8–81). Among the 33 carriers with available fundus data, 26/33 (78.8%) had fundus abnormalities. Carriers with abnormal fundi had poorer mean visual acuity than those with normal fundi (0.6 ± 0.5 vs. 0.1 ± 0.1 logMAR, *p* = 0.03). These carriers were younger on average than those with a normal fundus (mean age 40.6 vs. 54.1 years). Overall mean visual acuity was 0.5 logMAR (median 0.2, range: 0–1.9). In contrast to affected males, no significant correlation was found between logMAR visual acuity and age in female carriers, suggesting that visual acuity was not consistently declining with age in the carrier group [33]. Comander et al. likewise described clinical abnormalities in a high percentage of XLRP carriers; however, they did not present the clinical features of *RP2* carriers separately from *RPGR* carriers. They noted that there were no significant differences between *RP2* carriers and *RPGR* carriers with regard to mean final dark adaptation threshold elevation and mean ERG amplitude [34].

#### 3.3.2. Phenotypic Variability and Possible Mechanisms

Marked interocular and intrafamilial variability has been described in female *RP2* carriers. Lee et al. [35] described a female carrier of a heterozygous *RP2* frameshift variant who had extensive retinal degeneration and severe functional impairment in one eye, whereas the fellow eye was relatively preserved. Jayasundera et al. [20] reported one carrier with pronounced interocular asymmetry, including approximately 8 D of anisometropia. In the study of Sun et al. [32], one carrier showed striking interocular asymmetry, with bone‐spicule pigmentation and leopard changes in the posterior pole of the right eye but an almost normal appearance of the left eye. Pelletier et al. found marked intrafamilial variability among five female carriers of the same *RP2* frameshift variant (c.540_541delGT): Only one developed severe RP, beginning at approximately 30 years of age. This woman had an X‐autosome translocation, and the authors considered skewed X‐chromosome inactivation as a possible explanation. However, X inactivation could not be assessed in this individual [36].

These observations make X‐chromosome inactivation a plausible biological contributor to variable phenotypic expression in female carriers of pathogenic *RP2* variants. For an *RP2* heterozygous female, X‐chromosome inactivation creates a mosaic retina, with some photoreceptors expressing the normal *RP2* allele and others the mutant allele. If the proportion of cells with an active mutant X differs substantially between the two eyes, this could plausibly produce an asymmetric phenotype. However, skewed X‐inactivation patterns have not yet been systematically confirmed in *RP2* carriers with asymmetric disease presentation.

#### 3.3.3. Genotype–Phenotype Correlation

The available evidence does not establish a clear genotype–phenotype correlation in female carriers. In Georgiou et al., three of the four symptomatic carriers had null variants, whereas the remaining symptomatic carrier had a missense variant. However, carriers with null variants could also have a normal fundus, peripheral pigmentary changes, or a tapetal‐like reflex, arguing against a simple relationship between variant class and clinical phenotype [31]. Saeed et al. likewise found no significant association between visual acuity and mutation type (*p* = 0.97) or mutation location (*p* = 0.83) [33].

### 3.4. Genetic Variants in *RP2*

#### 3.4.1. Gene and Protein Structure

The *RP2* gene is located on the long arm of the X chromosome and contains five exons [37]. The largest transcript annotated on Ensembl corresponding to RefSeq sequence NM_006915.3 is the transcript ENST00000218340.4 which spans 3700 base pairs (bp), including 1053 bp of coding sequence (Figure 2A). *RP2* encodes a protein of 350 amino acids (RP2; XRP2, Uniprot ID O75695), which contains an N‐terminal tubulin cofactor C (TBCC)‐like and a C‐terminal nucleoside diphosphate kinase (NDPK)‐like domain [15, 38, 39], as well as N‐terminal myristoylation and palmitoylation sites (Figure 2B–D) [39, 40].

**Figure 2:**
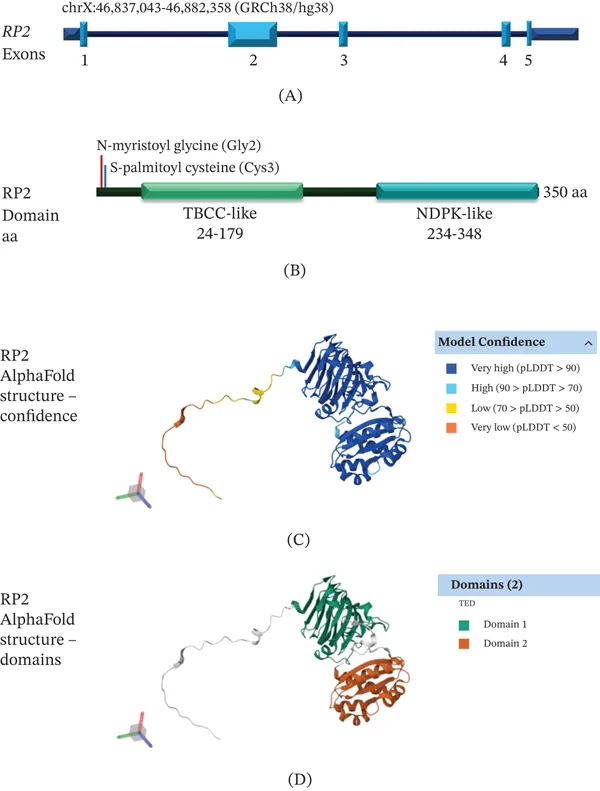
Schematic overview of the *RP2* gene and the RP2 protein. (A) Representation of five exons of *RP2* as light blue boxes and UTRs as darker blue boxes. Exon numbers and sizes according to ENST00000218340.4. (B) Representation of the 350 amino acid (aa) RP2 protein. The tubulin cofactor C (TBCC)–like domain and the nucleoside diphosphate kinase (NDPK)‐like domain are represented as light green boxes. Myristoylation and palmytoylation sites at amino acid Residues 2 and 3 are indicated by colored lines. Protein representation relies on PROSITE profile PS51374. aa, amino acid. (C) AlphaFold prediction AF‐O75695‐F1‐v6 of RP2 showing the predicted protein structure with axes oriented automatically. Color annotation shows model confidence (see legend from AlphaFold). Model creation date: Aug 1, 2025, https://alphafold.ebi.ac.uk/entry/AF-O75695-F1 accessed last on August 26, 2026, at 1:42 p.m. UTC+1. (D) AlphaFold prediction AF‐O75695‐F1‐v6 of RP2 showing the predicted protein structure with axes oriented automatically. Color annotation shows predicted TED domains (see legend from AlphaFold). Model creation date: August 1, 2025, https://alphafold.ebi.ac.uk/entry/AF-O75695-F1 accessed last on August 26, 2026, at 1:47 p.m. UTC+1.

#### 3.4.2. Genetic Basis of RP2‐Associated Retinopathy

*RP2* was identified as a disease gene for XLRP through positional cloning in 1998, establishing *RP2*‐associated disease as a distinct genetic form of XLRP [37]. Subsequent mutation‐screening studies identified pathogenic variants across the *RP2* coding region, including nonsense, frameshift, splice‐site, missense, and small deletion variants, and established a broad allelic spectrum among affected families, with a predominance of protein‐truncating variants [7, 8, 41–46]. Later studies further expanded the spectrum of reported *RP2* variants in different populations and cohorts and confirmed truncating variants as the main disease cause [27, 47–52]. Pathogenic variants have been identified across all five exons of *RP2*, although many reported missense variants are located within the TBCC‐like domain [37, 53, 54]. Recurrent variants include p.Arg118His and, most prominently, p.Arg120Ter, which has been frequently reported and described as a potential mutational hotspot [18, 19, 30, 55]. Moreover, whole gene deletions and intragenic deletions of *RP2* have been reported in a minority of patients [19].

#### 3.4.3. ClinVar Variant Analysis

Because several pathogenic missense variants have been reported to cluster within the TBCC‐like domain, with exon 2 described as a potential mutational hotspot [54, 56], we asked if the distribution of clinically identified *RP2* variants reveals a reproducible enrichment of variants in specific exons or whether the observed distribution can be explained by differences in exon size. To address this question, we analyzed clinically interpreted *RP2* variants retrieved from the ClinVar database [13] according to their variant type and genomic localization.

#### 3.4.4. Variant Types

We identified 325 relevant variants classified as ACMG/AMP Class 3 (variant of uncertain significance), Class 4 (likely pathogenic), or Class 5 (pathogenic). For this analysis each distinct nucleotide‐level variant was counted once based on VCV records, irrespective of the number of ClinVar submissions or reports, and variant counts were evaluated relative to exon size in bp, based on the canonical *RP2* transcript ENST00000218340.4 (NM_006915.3) [14].

We found that among Class 4 and 5 variants, the predominant variant types were frameshift (89/176) and nonsense (40/176) variants, together accounting for 73.2% of variants in these categories, followed by canonical splice‐site variants and initiator codon variants (Figure 3A). Additionally, one pathogenic deletion affecting the entire *RP2* gene with a size of 42,671 bp, associated with the phenotype “retinal dystrophy,” can be found in ClinVar (Variation ID: 3248692). These results indicate that causative variants in *RP2* are mostly associated with loss of function, which is in line with the literature [54].

**Figure 3:**
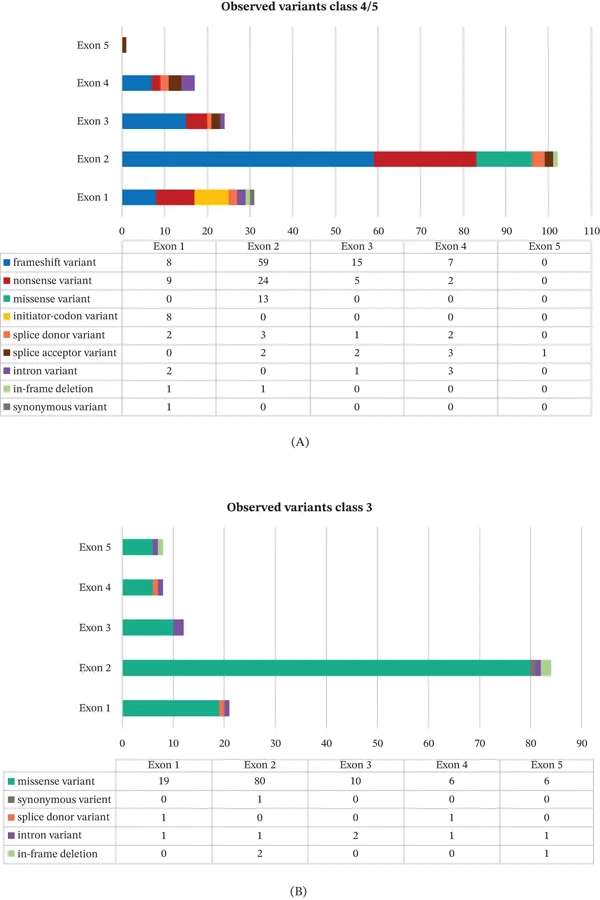
*RP2* variant types per exon. Clinically interpreted variants of ACMG (A) Classes 4 and 5 and (B) ACMG Class 3 were analyzed regarding variant type and exon localization based on ClinVar VCV records. Variants are distributed across the entire gene, with an apparent excess of variants in exon 2. Colors denote variant types: dark blue, frameshift; red, nonsense; green, missense; yellow, initiator codon; orange, splice donor; brown, splice acceptor; purple, intron variant; light green, in‐frame deletion; grey, synonymous variant. Figure data tables show variant counts by variant type per exon.

Among Class 3 variants, 81.2% are missense changes (Figure 3B), indicating an overrepresentation of missense variants in this group. Additionally, two variants in the 5 ^′^ UTR and 14 variants in the 3 ^′^ UTR were identified among the Class 3 variants, together representing 10.7% of ClinVar‐annotated *RP2* VUS.

#### 3.4.5. Exonic Variant Distribution

The large majority of ClinVar‐annotated variants is located in exon 2, consistent with its substantially larger coding length. We hypothesized that *RP2* variants are distributed across the five coding exons in proportion to exon length (null hypothesis). However, when observed variant counts were evaluated against the exon length, the distribution of ACMG Class 4/5 variants deviated significantly from expectation (Pearson′s *χ* ^2^ = 21.07, degrees of freedom (*d* *f*) = 4, *p* = 0.0003; *G* log − likelihood = 26.77, *d* *f* = 4, *p* = 0.00002; Monte Carlo *p* = 0.00046). Standardized‐residual analysis localized this deviation to two exons, both of which remained significant after Bonferroni correction: Exon 1 was enriched for variants, with 31 observed versus 18.6 expected (adjusted *p* = 0.020), whereas exon 5 was markedly depleted, with one observed versus 12.3 expected (adjusted *p* = 0.006). Together these exons accounted for 88.3% of the global *χ* ^2^ signal, whereas exons 2–4 did not differ significantly from expectation (Table 1).

**Table 1:** Variant density (variants/bp) per coding exon ACMG Classes 4 and 5.

Exon	Size bp	Observed *n*	Expected *n*	Density/kb (obs.)	Residual	Contribution to chi^2^	*p*	Adjusted *p*
1	112	31	18.61	276.79	+2.87	8.24	0.0041	0.0205
2	666	102	110.68	153.15	−0.83	0.68	0.4091	1.0000
3	115	24	19.11	208.70	+1.12	1.25	0.2635	1.0000
4	86	17	14.29	197.67	+0.72	0.51	0.4739	1.0000
5	74	1	12.30	13.51	−3.22	10.38	0.0013	0.0064
Total	1053	175	174.99	166.19	—	21.06	—	—

*Note:* UTR sequences were excluded from this analysis. Splice‐site variants were included with the respective adjacent exon. Exon sizes are based on Ensembl‐annotated transcript ENST00000218340.4. Values in expected *n*, density/kb, residual, and contribution on to chi^2^ columns are rounded to two decimal places. Adjusted *p* value is Bonferroni correction across five exon comparisons (adjusted *p* = max (*p*∗5, 1.000)).

A leave‐one‐variant‐type‐out sensitivity analysis indicated that these two signals are not equally robust. The exon 1 enrichment was particularly sensitive to the exclusion of initiator codon and nonsense variants and lost significance when either variant type was excluded (Table 2). This suggests that the exon 1 signal may reflect the concentration of these variant types near the start codon rather than a general elevation in variant rate across the exon. In contrast, the depletion in exon 5 persisted across nearly all exclusions, losing significance only when frameshift variants were removed (Table 2). However, this result needs to be interpreted cautiously because of the low number of variants (*n* = 1).

**Table 2:** Leave‐one‐variant‐type‐out analysis for ACMG Class 4/5.

Excluded variant type	Remaining variant counts	Global chi^2^ (*d* *f* = 4)	*p*	Exon 1 adjusted *p*	Exon 5 adjusted *p*
**Initiator codon**	**167**	**14.14**	**0.0069**	**1.000**	**0.009**
**Nonsense**	**135**	**14.96**	**0.0048**	**0.219**	**0.029**
In‐frame deletion	173	20.16	0.0005	0.034	0.007
**Frameshift**	**86**	**28.85**	**0.0000**	**0.000**	**0.201**
Synonymous	174	19.90	0.0005	0.038	0.007
Splice donor	167	18.72	0.0009	0.038	0.009
Splice acceptor	167	22.69	0.0001	0.008	0.003
Intron	169	18.07	0.0012	0.047	0.008
Missense	162	25.57	0.0000	0.005	0.010
**None (all variant type–baseline)**	**175**	**21.07**	**0.0003**	**0.020**	**0.006**

*Note:* UTR sequences and variants were excluded from this analysis. Splice‐site/intronic variants were included with the respective adjacent exon. Exon sizes are based on Ensembl‐annotated transcript ENST00000218340.4. Values in global chi^2^ column are rounded to two decimal places.

Variant distribution across exons of ACMG Class 3 variants did not differ significantly from the exon‐length expectation (Pearson′s *χ* ^2^ = 4.71, *d* *f* = 4, *p* = 0.32; *G* log − likelihood = 4.39, *d* *f* = 4, *p* = 0.35608; Monte Carlo *p* = 0.31609), and no individual exon showed a significant Bonferroni‐adjusted residual (Table 3). The leave‐one‐variant‐type‐out sensitivity analysis for this variant class did not change this result (Table 4).

**Table 3:** Variant density (variants/bp) per coding exon ACMG Class 3.

Exon	Size bp	Observed *n*	Expected *n*	Density/kb (obs.)	Residual	Contribution to chi^2^	*p*	Adjusted *p*
1	112	21	14.15	187.50	+1.82	3.31	0.0684	0.3421
2	666	84	84.12	126.13	−0.01	0.00	0.9896	1.0000
3	115	12	14.53	104.35	−0.66	0.44	0.5076	1.0000
4	86	8	10.86	93.02	−0.87	0.75	0.3851	1.0000
5	74	8	9.35	108.11	−0.44	0.19	0.6596	1.0000
Total	1053	133	133.01	126.30	—	4.69	—	—

*Note:* UTR sequences and variants were excluded from this analysis. Splice‐site/intronic variants were included with the respective adjacent exon. Exon sizes are based on Ensembl‐annotated transcript ENST00000218340.4. Values in expected *n*, density/kb, residual, and contribution on to chi^2^ columns are rounded to two decimal places. Adjusted *p* value is Bonferroni correction across five exon comparisons (adjusted *p* = max (*p*∗5, 1.000)).

**Table 4:** Leave‐one‐variant‐type‐out analysis for ACMG Class 3.

Excluded variant type	Remaining variant counts	Global chi^2^ (*d* *f* = 4)	*p*	Exon 1 adjusted *p*	Exon 5 adjusted *p*
Missense	12	5.12	0.2753	1.000	1.000
Splice donor	131	4.47	0.3466	0.521	1.000
Intron	127	5.80	0.2146	0.387	1.000
In‐frame deletion	130	5.21	0.2668	0.269	1.000
Synonymous	132	4.75	0.3138	0.316	1.000
None (all variant type‐baseline)	133	4.71	0.3186	0.342	1.000

*Note:* UTR sequences and variants were excluded from this analysis. Splice‐site/intronic variants were included with the respective adjacent exon. Exon sizes are based on Ensembl‐annotated transcript ENST00000218340.4. Values in global chi^2^ column are rounded to two decimal places.

Because the primary variant analysis counted each distinct nucleotide‐level variant only once and therefore did not capture information on repeated clinical submissions, we additionally reviewed variants with multiple ClinVar submissions to identify potentially recurrently reported variants. Among these variants, Codons 118 and 120 were notable for recurrently submitted changes. Five distinct nucleotide‐level variants affecting Codon 118 were identified, resulting in p.Arg118Gly, p.Arg118Cys, p.Arg118fs, p.Arg118Leu, or p.Arg118His. Three of these had multiple ClinVar submissions, with p.Arg118Cys (9 submissions) and p.Arg118His (8 submissions) showing the highest submission counts at this codon. The recurrent p.Arg120Ter variant had 12 ClinVar submissions, the highest number observed in the dataset. All other variants with multiple submissions had between two and four submissions (Table S5). Given the potential influence of reporting and ascertainment patterns, these observations are only presented descriptively and not interpreted as an objective representation of mutation frequency.

### 3.5. Current Knowledge on *RP2* Function

The *RP2* gene encodes the ubiquitously expressed RP2 protein, which consists of 350 amino acids [37, 57, 58]. RP2 is expressed throughout the human retina [58]. It is localized to the ciliary apparatus, which is the cilium‐associated centriole and the basal body of photoreceptor cells [40]. RP2 is both myristoylated and palmitoylated at the N‐terminus, and this dual posttranslational modification has been shown to target the protein to the plasma membrane [59], where it can bind to importin‐ß2 [60]. Importin‐ß2 binding is necessary to localize RP2 to the ciliary apparatus, where it is essential for maintaining ciliary structure and function and involved in the transport of prenylated proteins as well as G proteins such as transducin [40, 60–62].

RP2 is known to function as a GTPase‐activating protein (GAP) for the small GTPase ARL3, a ciliary protein that plays a critical role in the trafficking of proteins to the outer segments of photoreceptors [38, 62, 63] and has been implicated in an autosomal‐dominant form of RP and in autosomal‐recessive Joubert syndrome [64, 65]. Specifically, RP2 facilitates the conversion of active ARL3‐GTP to inactive ARL3‐GDP, thereby regulating the availability of ARL3 for its downstream effectors such as prenyl‐binding protein *δ* (PrBP*δ*) and UNC119 [61, 63].

RP2 not only localizes to the cellular membrane and the ciliary basal body but also to the periciliary ridge and the Golgi complex [40]. Its association to the Golgi is also reflected in dispersal of Golgi structures and, secondarily, of intraflagellar transport (IFT) proteins upon *RP2* knockdown in a cellular model [40]. This possibly implicates RP2 in the maintenance of Golgi membrane structure. It has been shown that ARL3 and RP2 can bind vesicle membranes and that the regulation of ARL3 by RP2 is important in vesicular traffic of proteins involved in phototransduction from the Golgi to the base of the cilium [40, 66]. Consistently, *RP2* knockout in mice has been shown to result in mislocalization of cone opsins to the nuclear and synaptic retinal layers prior to the onset of cone photoreceptor degeneration [56]. Failed vesicular transport of prenylated phosphodiesterase (PDE6) subunits and G‐protein‐coupled receptor kinase 1 (GRK1) to the outer segment in another murine knockout model supports the important role of RP2 in vesicular transport [62].

Furthermore, RP2 is implicated in the remodeling of the actin cytoskeleton through an interaction with OSTF1 [67, 68]. Actin remodeling is particularly relevant in the context of photoreceptor function as it contributes to the maintenance of the outer segment′s architecture [11]. Both RP2′s roles in actin skeleton organization and ciliary elongation may contribute to the morphological changes seen in cones from *RP2*‐knockout mice, displaying abnormal extension of the cone outer segment as well as ultrastructural abnormalities with disorganized lamellae [69, 70].

As in humans and mice, loss of RP2 function leads to retinal degeneration also in zebrafish [54, 67, 71, 72]. Zebrafish models have demonstrated that pathogenic variants in *RP2* predominantly result in the loss of protein stability [54, 67]. However, some missense variants affect the interaction with ARL3 or the binding to importin‐ß2, thus impairing the ability of RP2 to localize to the cilium [54, 60, 72]. Interestingly, some specific missense variants were found to differentially impact cone‐ and rod‐specific protein expression in zebrafish, which might relate to some of the phenotypic variability seen in humans [72]. Likewise, cellular experiments investigating the p.Gln158Pro variant, identified in a family with *RP2*‐associated retinopathy, reduced RP2 protein stability through increased ubiquitination and proteasomal degradation [51].

In addition, preliminary evidence suggests that RP2 function may extend beyond membrane dynamics, vesicle transport, and actin remodeling. Yoon et al. reported that RP2 exhibits exonuclease activity and translocates to the nucleus upon exposure to DNA‐damaging agents, including UVA radiation, potentially implicating it in base excision repair [73]. The diverse functions attributed to RP2 across cellular compartments and pathways, together with its ubiquitous expression pattern, suggest that RP2 may be essential not only for rods and cones but also for other retinal cell types, with potential therapeutic implications that warrant further investigation.

### 3.6. Therapeutic Advances

So far, few encouraging therapeutic strategies have emerged for *RP2*‐related RP, with gene addition therapy being supported by the strongest *RP2*‐specific preclinical evidence. Lane et al. developed *RP2* knockout and patient‐derived iPSC retinal organoids that replicated the photoreceptor degeneration phenotype. Strikingly, these organoids exhibited a peak in rod cell death by Day 150 of culture and ONL thinning by day 180. AAV2/5‐mediated *RP2* delivery under a CAG promoter rescued the degenerative phenotype by restoring rhodopsin expression and preserving ONL integrity, validating the model′s potential for preclinical testing [11]. Mookherjee et al. provided similar results using *RP2* knockout mice. Their study confirmed early‐onset cone dysfunction and progressive cone degeneration in the knockout mice, mirroring human *RP2* pathology. Gene addition via AAV8 carrying human *RP2* under a photoreceptor‐specific human rhodopsin kinase (hGRK1) promoter and using a self‐complementary vector genome configuration preserved cone function for up to 18 months. Crucially, cone opsin trafficking and cone PDE6 expression were restored, and benefits were observed even in late‐stage treatment. However, high‐dose administration induced retinal toxicity, especially in rods, emphasizing the need for precise dosing in future clinical trials [12]. Together, these studies support *RP2* gene addition as a potential therapeutic approach. However, its efficacy has not yet been tested in patients.

Another approach is to target the individual causative variant, for instance by genome editing or read‐through therapies. Schwarz et al. (2015) investigated translational read‐through inducing drugs (TRIDs) such as G418 and Ataluren (PTC124) in induced pluripotent stem cell (iPSC)‐derived RPE and retinal cells harboring the *RP2* NM_006915.3:c.358C>T p.Arg120Ter (R120X) nonsense variant. These compounds successfully restored full‐length RP2 protein and corrected aberrant intracellular trafficking, providing proof of concept for variant‐specific read‐through therapy [74]. Variant‐specific therapies might also be achieved through genome‐editing approaches. Genome editing approaches have not been applied in *RP2-*associated retinopathy so far but have shown promise in a cellular model of *RPGR-*associated retinal dystrophy [75]. However, experience with *RPGR* cannot be directly extrapolated to *RP2*, and the feasibility of this experimental approach has to be evaluated specifically for *RP2*.

In contrast to gene specific approaches, several pharmacological and nutritional approaches have been investigated for RP in general but not for *RP2-*associated retinopathy specifically. For instance, high‐dose vitamin A supplementation has historically been investigated in RP, but its efficacy and safety remain controversial [2]. Additionally, neuroprotection by drugs counteracting oxidative stress has received increasing interest [76, 77]. One compound under investigation is N‐acetylcysteine (NAC), an antioxidant which might protect photoreceptor cells against the negative impact of oxidative stress [78, 79]. In a Phase I trial in patients with RP, oral NAC was given at 600–1800 mg twice daily for 12 weeks, followed by the same dose three times daily [79]. Doses of 1200 mg and 1800 mg twice daily were safe and well tolerated, whereas gastrointestinal adverse events were more frequent at 1800 mg three times daily. The highest dose produced the most consistent improvement in cone sensitivity, but increasing the dosing frequency did not further raise aqueous NAC levels, suggesting a limit to intraocular availability. Although detectable aqueous concentrations indicate reasonable systemic absorption, the very high oral doses required likely reflect NAC′s low systemic bioavailability and rapid metabolism [80]. Thus, although oral NAC appears safe and achieves measurable ocular exposure, its pharmacokinetic profile suggests that more efficient delivery strategies or alternative thiol‐antioxidants may be needed for sustained retinal protection [78, 79]. NAC amide (NPI‐001), a lipophilic NAC‐derivative showing increased bioavailability and improved blood‐brain barrier permeability [81], is currently being investigated in a Phase I/II trial in Usher syndrome–related RP (NCT04355689). Additional neuroprotective compounds have been identified through high‐throughput drug screens and might potentially progress to clinical trials after further investigation [82].

Furthermore, cell‐based therapies using different kinds of stem cells, such as embryonic stem cells or iPSCs, are under investigation for different types of RP [83]. One recent study demonstrated safety and stable survival for 2 years of an allogenic iPSC‐derived retinal organoid sheet transplant in two human subjects with RP [84]. However, the clinical efficacy and long‐term outcomes of such techniques will have to be determined and there is not yet any evidence of a stem cell therapy tested specifically in patients with *RP2-*associated retinopathy.

Optogenetic therapies represent a gene‐agnostic approach to restoring visual function in advanced retinal degeneration. These strategies introduce light‐sensitive proteins, or opsins, into surviving retinal cells, such as remaining retinal ganglion or bipolar cells, thereby enabling them to respond directly to light despite the loss of functional photoreceptors [85, 86]. This approach is therefore potentially applicable across different genetic causes of inherited retinal degeneration and may be particularly relevant to advanced disease stages, in which conventional photoreceptor‐directed therapies are no longer feasible. Preclinical and early clinical studies in inherited retinal degenerations have provided proof of concept for optogenetic restoration of light sensitivity and visual function [86–88]. Although these studies are not specific to *RP2-*associated retinopathy, they provide a rationale for considering optogenetics as a potential future therapeutic strategy for patients with advanced *RP2-*associated retinopathy.

## 4. Discussion

Although a certain degree of clinical variability has been reported for *RP2-*associated retinopathy, the disease course typically shows childhood onset, with nyctalopia as the most common initial symptom. It is characterized by symmetrical bilateral disease with early peripheral vision loss and early and progressive decline of central vision. Although generally classified as a form of RP, its degeneration pattern often deviates from the classical RP sequence of rod‐predominant loss with subsequent cone involvement. Several studies have noted that some individuals with *RP2-*associated retinopathy show a cone‐rod dystrophy–like or mixed functional pattern, challenging strict classification as RP [19, 20]. Accordingly, recent literature defines *RP2*‐related retinopathy as a rod‐cone dystrophy [89].

Given the early onset and rapid progression of *RP2-*associated retinopathy [18, 19], often affecting central vision early in the disease course, regular clinical monitoring appears appropriate. No validated clinical monitoring protocol currently exists for *RP2-*associated retinopathy. Therefore, based on the available observational evidence and our clinical experience, we propose an individualized approach to clinical follow‐up. At diagnosis, baseline assessment should include BCVA, OCT, FAF, full‐field ERG, PERG, and wide‐field fundus photography. Younger patients may benefit from regular follow‐up every 4–6 months to monitor visual decline and potentially treatable complications such as cystoid macular edema. Supportive care and visual rehabilitation should be offered as needed. Based on the variable findings in female carriers [27, 31], we propose a similarly stratified approach to monitoring. Baseline assessment should include multimodal imaging and ERG testing. Asymptomatic carriers without detectable abnormalities may be monitored every 2–3 years, whereas those with subtle signs, such as FAF changes or mild ERG abnormalities, may be examined annually. Symptomatic carriers or those with progressive changes on OCT or ERG may warrant more frequent follow‐up every 6–12 months. We propose that a clinical monitoring protocol should be prospectively validated in a cohort of *RP2*‐associated retinopathy.

From a medical geneticist′s perspective, we argue that children presenting with nyctalopia should receive comprehensive genetic testing early on to avoid diagnostic uncertainty and to guide patient management. Next‐generation‐sequencing (NGS)–based gene‐panel testing can be a reliable and cost‐effective first‐line test, usually yielding a high diagnostic rate of approximately 70% or higher in patients with RP, although diagnostic yield varies substantially with cohort composition, inheritance pattern, and age at onset [90–92]. In XLRP, NGS panel diagnostics may achieve yields higher than 85% [90]. We suggest that in unsolved cases, short‐read or long‐read genome sequencing should be considered to identify complex structural aberrations, noncoding variants, or novel disease‐associated genes.

Our analysis of 325 distinct clinically interpreted *RP2* variants provides a quantitative assessment of their distribution across the five exons. Because recurrently reported variants were counted only once at the nucleotide level, the analysis was not influenced by the number of ClinVar submissions or reports associated with individual variants. Overall, the distribution of Class 3 variants was consistent with the null expectation based on exon size, with no evidence of preferential enrichment or depletion. In contrast, the distribution of pathogenic and likely pathogenic variants (ACMG/AMP Classes 4 and 5) showed deviations from this expectation, with an enrichment of variants affecting the initiator codon in exon 1 and a depletion of variants in exon 5. Sensitivity analysis showed that the apparent enrichment in exon 1 was almost entirely attributable to initiator codon variants, whereas the depletion observed in exon 5 was not readily explained by any variant category. The basis for the apparent depletion of Class 4/5 variants in exon 5 remains uncertain and may reflect biological constraint, ascertainment bias, or differences in variant classification. However, the number of reported variants in this category does not permit a definitive interpretation of this observation.

The variant classes also differed markedly in their molecular composition. Of the 149 Class 3 variants, 121 (81.2%) were missense variants, whereas pathogenic and likely pathogenic variants (ACMG/AMP Classes 4 and 5) were predominantly truncating variants (Table S5). The predominance of missense variants among Class 3 variants highlights the challenge of interpreting missense variation, for which available evidence may be insufficient to distinguish pathogenic from benign variants using the ACMG/AMP framework [14]. Because VUS do not translate into clinical decision‐making, the high burden of missense variants in this variant class may contribute to unresolved diagnoses. Addressing this gap requires systematic generation of functional evidence and structured approaches to variant interpretation and reclassification for *RP2*. Although long‐read RNA sequencing in patient‐derived fibroblasts can identify aberrant splicing events, this approach is expected to resolve only a subset of variants. Complementary strategies targeting protein stability, subcellular localization, and interactions with known binding partners may provide additional evidence, ideally in disease‐relevant cellular systems [72, 93]. We are currently developing a systematic pipeline for improved bioinformatics and functional assessment of *RP2* missense variants to help establish genotype–phenotype relationships and improve patient selection for future therapeutic studies [94].

Because the primary analysis was based on single counts of distinct ClinVar variants, it does not capture information on recurrently submitted variants. Review of variants with multiple ClinVar submissions identified several variants that were repeatedly reported, with a notable concentration at Codons 118 and 120 within the TBCC‐like domain of RP2. At Codon 118, five distinct nucleotide‐level changes were identified, all affecting the same arginine residue, three of which were repeatedly submitted to ClinVar. Notably, four of the variants identified at Codon 118 (p.Arg118His, p.Arg118Gly, p.Arg118Leu, and p.Arg118Cys) have previously been functionally investigated. These variants do not substantially affect RP2 protein abundance, and p.Arg118Gly was shown to markedly reduce RP2–ARL3 interaction, whereas previous studies demonstrated impaired ARL3 binding for p.Arg118His [53, 54, 95]. The p.Arg120Ter variant represented the most frequently submitted variant in this analysis, with 12 submissions, which is in keeping with the literature, where this variant has been repeatedly reported multiple times within cohorts [18, 19, 44, 55, 96]. However, ClinVar reflects clinical testing, variant interpretation, and reporting practices that may differ between laboratories. Therefore, submission counts do not necessarily reflect the number of independent individuals or families carrying a given variant. Thus, the observations regarding multiple submissions in ClinVar as well as the observed variant distribution may be influenced by bias of ascertainment and reporting. Determining whether these observations reflect the underlying distribution of *RP2* variation, recurrent variants, or even mutational hotspots will require systematic clinical and genetic data from well‐characterized patients and families. In parallel, systematic clinical characterization is required to connect genetic findings with disease manifestations and to establish sufficiently well‐characterized cohorts for translational research. To advance this goal, the InsightRP2 registry was established to collect genetic, clinical, and retinal imaging data from individuals with *RP2*‐associated RP, in order to support natural‐history studies, genotype–phenotype analyses, and the development of trial‐ready cohorts [94].

A number of preclinical and early‐phase clinical studies indicate potential for both gene‐specific and gene‐agnostic strategies in RP. AAV‐mediated gene addition has shown durable rescue of photoreceptor structure and function in animal and cellular models [11, 12], and small‐molecule approaches are also under investigation to modulate disease pathways [78, 79, 82]. Importantly, gene addition therapy has already progressed to clinical evaluation in XLRP because of *RPGR* variants, where safety and efficacy data are emerging [97], suggesting that similar approaches for *RP2* might be technically feasible. Such strategies appear particularly promising because of the relatively small size of the *RP2* coding sequence, which can be accommodated by standard AAV vectors and applied independently of the underlying pathogenic variant [11]. Experience from *RPGR* gene therapy provides an important reference for promoter and targeting strategies for *RP2* gene addition. Current *RPGR* trials typically use the photoreceptor‐specific hGRK1 promoter, reflecting the predominant photoreceptor phenotype and the strong enrichment of RPGR in the photoreceptor connecting cilium [97–99]. Because *RP2-*associated retinopathy also presents primarily as a photoreceptor disease, photoreceptor‐targeted promoters represent a logical starting point for *RP2* gene therapy, also minimizing the risk of ectopic expression–induced toxicity. However, expression of *RP2* in other retinal cell types such as Müller, horizontal, and amacrine cells, and the RPE [100], as well as the diverse functions attributed to RP2 [39, 40, 61, 73, 101, 102], raise the possibility that RP2 may contribute to retinal homeostasis in multiple cell types, not solely photoreceptors, particularly over the long periods relevant to the human disease course. Moreover, it is currently unknown whether the progressive loss of the RPE documented in affected individuals [18, 19] is a secondary phenomenon related to photoreceptor death and consecutive inflammation or whether it derives directly from loss of RP2 function in epithelial cells. Because of these considerations, strategies and concepts from *RPGR* related studies cannot be directly transferred to *RP2* therapeutic development. Target cells and delivery route need to be determined specifically for potential *RP2*‐AAV constructs. Importantly, preclinical studies on *RP2* have shown that both photoreceptor‐specific promoters and ubiquitous promoters achieve effective *RP2* rescue in mouse models and human retinal organoids and that toxicity has been primarily dose‐dependent, without clear evidence that promoter restriction alone mitigates risk [11, 12]. Thus, systematic, dose‐matched comparison of photoreceptor‐restricted versus broader promoter strategies will be important to define the optimal cellular targeting strategy for durable and safe *RP2* gene addition. On the same line, once the target cell population is defined, capsid engineering strategies can be explored to increase efficacy of target cell transduction and to decrease off‐target transduction [103]. This strategy yielded already in a number of promising novel capsid variants for retinal gene therapy such as AAV‐7m8, AAV‐NN or AAV‐GL [104, 105].

Additionally, variant‐specific therapies, based on genome editing strategies, will need to be further investigated [106]. For *RP2*, targeted repair of single nucleotide variants by base editing or repair of indels and complex splice‐site variants by prime editing might present interesting therapeutic strategies. Such strategies have shown promising results in mouse models of different types of IRD [71, 107–113]. However, clinical application of these approaches may be limited by the high number of individual genetic variants in the *RP2* gene, necessitating the production of medications tailored specifically to each patient.

At advanced stages of *RP2*‐associated RP, when most photoreceptors have degenerated and gene addition or repair strategies might no longer be effective, optogenetic therapy might someday offer a therapeutic option, by making the remaining inner retinal cells, such as bipolar or ganglion cells, directly light‐sensitive through introduction of specially engineered proteins (opsins) [114]. In recent years, new optogenetic tools such as red‐shifted channelrhodopsins (e.g. ChReef) have been developed that respond well under normal light conditions, and studies in blind mouse models have shown that this can restore meaningful visual responses [86]. For example, the optogenetic therapy MCO‐010 targets bipolar cells, taking advantage of their abundance and localization in the retina potentially enabling enhanced visual acuity and photosensitivity, thus eliminating the need for external light‐enhancing goggles [88]. However, these developments are at preclinical or early clinical stages and do not represent approved treatment options to date.

To advance gene‐based therapies for *RP2-*associated retinopathy toward clinical application, studies in patient‐derived organoids and relevant animal models will be essential for refining vector design, optimizing dosing strategies, and determining safety parameters as well as the optimal therapeutic window. A further key requirement for future *RP2* therapy trials is the careful definition of clinical endpoints and the establishment of well‐characterized patient cohorts to ensure trial readiness. Commonly employed outcome measures in IRD trials include BCVA, OCT‐derived structural markers, perimetry, ERG, and FAF [115]. Among more recent functional endpoints, low‐luminance visual acuity (LLVA) merits particular attention as a potentially sensitive metric. LLVA probes visual performance under mesopic conditions and complements standard photopic acuity, which can remain relatively preserved until later disease stages [115, 116]. In *RPGR*‐associated rod‐cone dystrophy, LLVA has proven sensitive to early functional decline and correlates well with OCT‐based structural changes [116, 117]. Microperimetry also represents a robust and sensitive measure, capable of capturing localized functional loss and widely adopted in *RPGR* gene‐therapy studies [118–120]. However, for *RP2-*associated retinopathy, no systematic LLVA and microperimetry datasets exist in the literature to date. Thus, generating such datasets will be essential for defining reliable, sensitive functional endpoints tailored to *RP2*. In this context, dedicated patient registries and structured natural‐history programs are urgently needed to overcome the limitations of very small cohort sizes and to support the development of clinically well‐defined, trial‐ready populations across different disease stages [94].

Emerging artificial intelligence (AI)–based analytic tools, particularly AI‐assisted OCT analysis, may further enhance clinical trial readiness by improving progression monitoring, refining inclusion criteria, and supporting the development of objective endpoints [121]. Recent deep‐learning studies in inherited retinal diseases, although not focused specifically on *RP2*, provide important methodological precedent. Liu et al. demonstrated that multimodal imaging (IR and OCT) can be used to predict visual impairment in RP, showing the feasibility of learning structure–function relationships directly from retinal images [122], an approach that is particularly relevant for *RP2*, where early macular involvement is common. Further, Pontikos et al. also used multimodal imaging (FAF, infrared (IR) fundus, and OCT) to predict causal IRD genes, including *RP2*, highlighting how deep learning can capture subtle, gene‐associated phenotypes [123]. These studies illustrate that current deep‐learning methods can successfully extract functional and genetic signals from retinal imaging, underscoring their potential value for future *RP2*‐focused clinical trial designs.

## 5. Conclusions and Future Perspectives

*RP2-*associated retinopathy is an early‐onset retinal dystrophy that typically leads to complete vision loss by the third or fourth decade of life. Patients should undergo comprehensive evaluation by retinal dystrophy specialists and receive regular follow‐up. Early molecular genetic testing is crucial for diagnosis and clinical management. Clinically applicable functional assays are needed to resolve variants of uncertain significance, thereby improving diagnostic yield and reducing uncertainty and disadvantages for patients who might otherwise be exempt from emerging therapeutic options. Gene addition therapy for *RP2-*associated retinopathy appears within reach; however, regulatory approval, funding, and recruitment of sufficient patient cohorts remain challenging. To address these barriers, we have initiated the InsightRP2 project (www.insight-rp2.org), a multidisciplinary effort integrating multiomics approaches, molecular and bioinformatic techniques, and clinical expertise with a dedicated, gene‐specific patient registry [94]. These combined efforts are aimed at facilitating the transition from preclinical research to clinical studies in *RP2-*associated retinopathy.

## Author Contributions

Nina Bögershausen performed the literature review, designed the figures, and wrote the manuscript, with contributions, ideas, and comments from all authors. Rozhin Bayati performed and interpreted statistical analyses. Maximilian Gerhardt contributed patient images and clinical information for Figure 1. All authors are members of the InsightRP2 consortium and contributed to the conception and completion of this article. Bernd Wollnik is the study PI.

## Funding

This study was funded by the Deutsche Forschungsgemeinschaft (10.13039/501100001659, EXC 2067/1‐390729940), the Deutsches Zentrum für Herz‐Kreislaufforschung (10.13039/100010447), and the Else Kröner Fresenius Center for Optogenetic Therapies Göttingen. Open Access funding enabled and organized by Projekt DEAL.

## Conflicts of Interest

The authors declare no conflicts of interest.

## Supporting Information

Additional supporting information can be found online in the Supporting Information section.

## Supporting information


**Supporting Information 1** Table S1: PubMed search terms.

**Supporting Information 2** Table S2: Relevant clinical and genetic studies of *RP2*‐associated retinopathy.

**Supporting Information 3** Table S3: Clinical findings in male individuals with *RP2*‐associated retinopathy.

**Supporting Information 4** Table S4: Clinical findings in female carriers of *RP2*‐associated retinopathy.

**Supporting Information 5** Data S1: Python code for statistical analysis of ClinVar annotated *RP2* variants.

## Data Availability

Details on governance, consent, inclusion criteria, and data‐protection procedures concerning the InsightRP2 registry are available on ClinicalTrials.gov. The study was approved by the ethics committee of the University Medical Center Göttingen (11/11/24). For more information on the InsightRP2 project and the patient registry, please visit the project website at www.insight-rp2.org. Figure 1A was created in BioRender by Bögershausen, N. (2026), with license to publish: https://BioRender.com/6cuht6l. Data spreadsheets and statistics code are available in the Supporting Information.
