# Supplementary material for: Insights Into RP2‐Associated Retinopathy: From Molecular Basis to Clinical Translation
Source: Hum Mutat. 2026 Oct 17;2026:1839164. doi: 10.1155/humu/1839164 (PMC13676036; doi:10.1155/humu/1839164)
Supplement: Supplementary file 1 [file HUMU-2026-1839164-s002.docx]

**Supplemental Table 4. Clinical findings in female carriers of *RP2*-associated retinopathy**

| Study | N | Age | Symptoms / VA | Fundus / OCT | ERG / VF /FAF | Additional findings |
| --- | --- | --- | --- | --- | --- | --- |
| Rosenberg et al., 1999 | 7 | NR | Family RP200307: onset < 18 (nyctalopia) in 1/3, and > 30 y in 2/3. Slow progression of VA loss.  Family RP200309: VA 6/18 to 6/60 when first measured (age 7 to 57 y). | Fundus: Family RP200307: central or peripapillary atrophy of the RPE and the CCL or both; Peripheral spicule hyperpigmentations scarce.  Family RP200309: Tongue-shaped peripapillary chorioretinal atrophies, diffuse and patchy atrophy, scattered peripheral hyperpigmentations, constricted arteries. | Family RP200307: ERG: subnormal or extinct in 2/3; VF: central scotomas in 3/3.  Family RP200309: ERG: Unrecordable at ages 28 and 37 in 2/4, subnormal in 1/4 at 39 y. Constricted VF in 1/4 and ring/paracentral scotomas in 2/4. | Family RP200307: High myopia (-20.0 and -10.5 D) in 1/3.  Family RP200307: CME in 2 carriers. Myopia up to – 16 D in 1/4. |
| Jayasundera et al. 2010 | 3 | Age at last exam 22 – 45 y | Poor VA in 2/3. Childhood onset in 1/3. | Fundus: macular atrophy in 3/3; marked interocular asymmetry in 1/3. | ERG: severely reduced photopic and scotopic responses in 2/3.  VF: central scotoma 2/3 | Anisometropia in 1/3. Longitudinal follow-up data for 2/3. |
| Kurata et al. 2019 | 5 | Median 58 y (47–73) | Visual disturbance 2/4 with available symptom data. Median decimal BCVA 0.9 (0.2–1.5). | Fundus: grade 0 2/8 eyes, grade 1 4/8, grade 2 2/8, grade 3 0/8. FAF: radial AF in 8/8 eyes.  OCT normal in the majority of carriers (no exact denominators given). | DA 3.0 ERG: normal 2/8, subnormal 2/8, reduced 4/8; no non-detectable responses. VF: median V-4e 21,253 mm² (18,339–22,501); I-4e 10,113 mm² (8,464–13,711). | - |
| Saeed et al. 2023 | 35* | Mean 44.2 y (8–81) | Mean VA 0.5 logMAR (median 0.2; range 0–1.9). ≥ 20/20 in 8/35; ≥ 20/25 in 11/35; ≥20/50 in 18/35; ≥20/200 in 30/35. | Fundus: abnormal in 26/33 (78.8%). | NR | Mean spherical equivalent −3.5 D (median −1.7, range +4.3 to −24.0). Anisometopia in 38% (8/21).  Meta-analysis. |
| Lee et al. 2023 | 1 | 25 y | 20/100 in OD and 20/20 in OS. | Fundus: bone spicule pigmentations, retinal vessel attenuation, tessellated fundus changes in OD; OS almost normal.  OCT: visible thinning of the ONL and foveal anomalies, small area of preserved EZ in OD; localized attenuation and EZ loss in OS. | ERG: extinguished rod response and severely impaired cone response in OD, normal in OS.  VF: a central visual field within 10° and central scotoma on OD, normal on OS.  FAF: diffuse, blotchy, or mottled hyperfluorescence in OD; OS normal | Case report of an affected female with marked interocular asymmetry |
| Georgiou et al. 2024 | 27 | no complaints: 42 y (16–76); symptomatic: 41 y (30–51), | 4/27 symptomatic with nyctalopia; 23/27 no visual complaints.  VA normal in 24/27, 3 symptomatic females had reduced VA which deteriorated with age. | Fundus (n=27): normal 8/27, TLR 10/27, scattered pigmentation 5/27, RP changes 4/27.  OCT (n=10): RP patients: atrophic and myopic changes with EZ loss; patients with TLR: intact retinal layers, increased reflectivity of RPE/EZ. | ffERG (n=11): 1/11 bilaterally undetectable, 2/11 normal, remainder mild–severe DA/LA attenuation. PERG: normal 6/11, unrecordable 1/11, subnormal 2/11, marginal abnormalities in 2 subjects.  FAF (n=11): women with TLR: radial FAF pattern. Patients with RP: variable degrees of midperipheral retinal degeneration in an asymmetric pattern. | Most comprehensive study of female phenotype based on pedigrees reported in Georgiou et al. 2023 and additional affected females. |
| Sun et al. 2026 | 5 | Av. 35.5 y (25–54) | BCVA: reduced in 8/10 eyes (logMAR 0.397–0.097). | Fundus: tessellation 7/10, hyperpigmentation 6/10, vessel attenuation 6/10, retinal atrophy 2/10. Asymmetric presentation in 1 carrier.  OCT: focal outer-retinal layer disruption reported in 7/10 eyes. | ERG: abnormal 8/10 eyes. Mean DA a/b-wave amplitudes 109.84 ± 92.09 / 239.38 ± 217.19 µV; LA a/b-wave amplitudes 27.45 ± 22.39 / 85.17 ± 56.35 µV. | Refraction: myopia in 7/10 eyes (average: −1.75 D, range: −4.375 D to 1.25 D).  Phenotype resembling that of affected males in 3/5 women. |

**Abbreviations**: AF, autofluorescence; Av, average; BCVA, best-corrected visual acuity; CCL, choriocapillary layer; CME, cystoid macular edema; D, diopter; DA, dark-adapted; ERG, electroretinography; EZ, ellipsoid zone; FAF, fundus autofluorescence; ffERG, full-field electroretinography; LA, light-adapted; NR, not reported; OCT, optical coherence tomography; OD, oculus dexter; OS, oculus sinister; PERG, pattern electroretinography; RP, retinitis pigmentosa; RPE, retinal pigment epithelium; TLR, tapetal-like reflex; VA, visual acuity; VF, visual field; VI, visual impairment; V-4e/I-4e, Goldmann visual-field areas using V-4e/I-4e stimuli; Y, years.
