# Supplementary material for: Insights Into RP2‐Associated Retinopathy: From Molecular Basis to Clinical Translation
Source: Hum Mutat. 2026 Oct 17;2026:1839164. doi: 10.1155/humu/1839164 (PMC13676036; doi:10.1155/humu/1839164)
Supplement: Supplementary file 2 [file HUMU-2026-1839164-s003.docx]

**Supplemental Table 3. Clinical findings in male individuals with *RP2*-associated retinopathy**

| Study | N | Age / onset | BCVA | Fundus / OCT | FAF | ERG / VF | Comments |
| --- | --- | --- | --- | --- | --- | --- | --- |
| Ponjavic et al. 1998 | 3 | 17-30 y at last examination.  Onset at 3 y of age. | BCVA: 0.7 or better in 3/3 | Fundus: bone-spicule pigmentation, vessel attenuation and disc pallor; macula remained normal in 3/3 | NR | ERG: Rod response non recordable, combined response severely reduced in 3/3.  VF: progressive constriction in 3/3 | Cataracts in 3/3 |
| Sharon et al. 2003 | 16 | Mean age 27.0 ± 4.3 y | Age-adjusted geometric mean BCVA 20/210 | NR | NR | 30-Hz ERG (n=13): 1.93 mV (normal ≥50 mV).  VF (n=15): 3,640 deg² (normal ≥11,399 deg²) | - |
| Jayasundera et al. 2010 | 15 | Age at exam 3-69 y.  Age of onset 2-25 y. | BCVA: worse than 20/50 before age 12 in 9/11 | Fundus: macular granularity/atrophy/bull's-eye 11/12 (92%); macular changes before age 12 in 10/11 (91%) | NR | ERG: severe rod-cone dysfunction 9/10 (90%), cone-rod pattern 1/10. VF: central scotoma 5/10; I4e constriction 6/8 <12 y | Severe vision loss (< 20/200) by the third to seventh decade of life |
| Kurata et al. 2019 | 6 | Median age 30 (17–42); median onset 7.5 y (3–11) | Median decimal BCVA 0.03 (CF–0.3) | NR | NR | VF: median V-4e 10,814 mm² (5,528–20,048); I-4e 3 mm² (0–52); V-4e declined with age. | Median refractive error -5.8 D (-10.0-+1.0). |
| Fujinami et al. 2020 | 4 (17) | Mean age at examination 31.2 (16–61) y (n=16); Mean onset 5.75 (3–11) y (n=8) | Mean BCVA OD  1.14 (0.70–1.52) and OS 1.25  (0.52–1.70) LogMAR (n=12). | OCT (n=4): diffuse photoreceptor-layer loss 3/4; relative foveal preservation 1/4. Fundus phenotype variable, including peripheral or posterior-pole predominant atrophy. | NR | VF (n=4): peripheral loss + central scotoma 2/4; complete VF loss 1/4; large central scotoma with preserved periphery 1/4 | Mean of refractive  error −6.6 D  (−12.0–0.75) OD and −6.1 D (−10.0–0.50) OS (n=10). |
| Cheloni et al. 2022 | 47 | Median age at last visit 27 y (n=41). Median symptom onset 7 y (IQR 2.25–12), n=14 | BCVA longitudinal (n=41): median 0.66 (IQR 0.35-1.4) → 1.3 (IQR 0.6-1.4) logMAR | Fundus (n=37): pigmentation 33/37 (89.2%), vessel attenuation 35/37 (94.6%), disc pallor 10/37 (27.0%), macular changes 22/37 (58.5%).  OCT (n=27): severe EZ disruption 23/35 (65.7%). | FAF (n=38): Severe hypo-AF 21/38 (55.3%), hyper-AF ring 9/38 (23.8%), central hyper-AF 6/38 (15.8%), peripheral granular-mottled pattern of hypo-AF in 2/38 (5.3 %). | ERG and VF available only in minority, severe loss reported, not analyzed in detail. | Estimated BCVA loss 46–49%/decade and CRT loss 12.6%/decade.  Cataract in 36.4% and pseudophakia in 22.7%. Median spherical equivalent −7.7 D (IQR: −8.3 to −6.2) (n=10). |
| Georgiou et al. 2023 | 54* | Onset (n=48): childhood 44/48 (91.7%), mean 7.6 ± 4.1 y; adult 4/48 (8.3%), mean 32.5 ± 16.2 y | Symptom onset: nyctalopia 33/48 (68.8%), reduced central VA 8/48 (16.7%), both 4/48 (8.3%).  BCVA: mean 0.91 / 0.94 → 1.17 / 1.16 logMAR (OD/OS) | OCT (n=46): complete EZ/ONL loss at mean 36.4 y in 23/42 with childhood onset; measurable EZ and ONL in 19/42. Longitudinal data (n=16): EZ width decrease of 219 µm/y and ONL decrease 4.93 µm/y. No identifiable EZ after age 26 y in childhood onset group. Stable imaging in 2/4 patients with late onset. | FAF (n=46): normal 11/46 (23.9%); diverse pattern ranging from paracentral macular ring, macular ring and midperipheral patchy changes to patchy macular signal with normal periphery. Macular changes in 31/46 (67.4%). | ERG (n=22): ffERG/PERG: rod-cone dystrophy pattern 17/22; ffERG undetectable in 3/22; ffERG responses consistent with severe rod and cone dystrophy in 2/22; PERG P50 undetectable in 20/22.  VF: not systematically reported. | Mean 7.3 y longitudinal follow-up on BCVA and 5.3 y for OCT; |
| Choi et al. 2024 | 2 | Symptom onset 4 y | NR | OCT: diffuse retinal thinning and EZ disruption 2/2 | NR | ERG: nearly flat responses at 14 and 16 y | Family report |
| Sun et al. 2026 | 6 | Age 1- 8 y | BCVA (n=3): mean logMAR value of 0.5235  (range: 0.301–0.698) | Fundus: pigmentary abnormalities,  tessellated changes, and yellowish-white punctate exudations.  OCT (N=4): thinning and / or disorder of ONL. | NR | ERG (n=4) decreased a-wave (average: 20.90 ± 17.05 μV and  10.76 ± 6.50 μV) and b-wave (average: 28.33 ± 21.59 μV and  22.01 ± 16.58 μV) amplitudes. VF: NR | Average spherical equivalent of −4.15 D  (range: −3.125 D to −6.25 D) |

**Abbreviations**: BCVA, best-corrected visual acuity; CF, counting fingers; CME, cystoid macular edema; CRT, central retinal thickness; D, diopter; DA, dark-adapted; ERG, electroretinography; EZ, ellipsoid zone; FAF, fundus autofluorescence; ffERG, full-field electroretinography; I4e/V4e, Goldmann visual-field stimuli; LA, light-adapted; NR, not reported; OCT, optical coherence tomography; OD, oculus dexter; ONL, outer nuclear layer; OS, oculus sinister; PERG, pattern electroretinography; RP, retinitis pigmentosa; VA, visual acuity; VF, visual field; Y, years.
