# Supplementary material for: Insights Into RP2‐Associated Retinopathy: From Molecular Basis to Clinical Translation
Source: Hum Mutat. 2026 Oct 17;2026:1839164. doi: 10.1155/humu/1839164 (PMC13676036; doi:10.1155/humu/1839164)
Supplement: Supplementary file 3 [file HUMU-2026-1839164-s004.docx]

| **Category** | String |
| --- | --- |
| **Clinical phenotype and natural history** | ("RP2" [Title/Abstract]) AND (retinopathy [Title/Abstract] OR retinitis [Title/Abstract] OR "retinal degeneration" [Title/Abstract] OR "visual acuity" [Title/Abstract] OR OCT [Title/Abstract] OR ERG [Title/Abstract]) |
| **Female carriers** | ("RP2" [Title/Abstract]) AND  (carrier* [Title/Abstract] OR female [Title/Abstract] OR woman [Title/Abstract] OR women [Title/Abstract] OR "X chromosome inactivation" [Title/Abstract] OR "X-inactivation" [Title/Abstract]) |
| **Genetics and genotype–phenotype correlations** | ("RP2" [Title/Abstract]) AND (retinopathy [Title/Abstract] OR retinitis [Title/Abstract] OR "retinal degeneration" [Title/Abstract]) AND (variant* [Title/Abstract] OR mutation* [Title/Abstract] OR "genotype-phenotype" [Title/Abstract]) |
| **Molecular and cellular function** | ("RP2" [Title/Abstract]) AND  (function [Title/Abstract] OR "molecular function" [Title/Abstract] OR cilia [Title/Abstract] OR cilia* [Title/Abstract] OR ARL3 [Title/Abstract] OR photoreceptor* [Title/Abstract] OR trafficking [Title/Abstract] OR "protein trafficking" [Title/Abstract]) |
| **Experimental models** | ("RP2" [Title/Abstract]) AND  (mouse [Title/Abstract] OR mice [Title/Abstract] OR zebrafish [Title/Abstract] OR cellular [Title/Abstract] OR iPSC [Title/Abstract] OR "stem cell" [Title/Abstract] OR organoid* [Title/Abstract]) |
| **Therapeutic approaches** | ("RP2" [Title/Abstract]) AND (eye [Title/Abstract] OR retin* [Title/Abstract]) AND (therap* [Title/Abstract] OR treatment [Title/Abstract] OR "read-through" [Title/Abstract] OR "replacement" [Title/Abstract] OR "stem-cell*" [Title/Abstract] OR optogenetic [Title/Abstract]) |

**Supplementary table 1. PubMed search terms**
