# Supplementary material for: Insights Into RP2‐Associated Retinopathy: From Molecular Basis to Clinical Translation
Source: Hum Mutat. 2026 Oct 17;2026:1839164. doi: 10.1155/humu/1839164 (PMC13676036; doi:10.1155/humu/1839164)
Supplement: Supplementary file 4 [file HUMU-2026-1839164-s005.docx]

**Supplemental Table 2. Relevant clinical and genetic studies of *RP2*-associated retinopathy**

| Study | Study type | Country | Cohort size | Sex | Genotype information | Main findings |
| --- | --- | --- | --- | --- | --- | --- |
| Schwahn et al., 1998 | Gene identification | Germany / Europe | 38 XLRP families | M | First description of p.Arg118His variant. Six variants in exons 1 or 2. One deletion of exon 4. | Initial identification of *RP2* as a disease gene for X-linked RP. Description of seven distinct RP2 variants. |
| Ponjavic et al., 1998 | Family-based study | Sweden | 1 family / 3 affected | M | Familial *RP2* genotype determined by haplotypes. | Early description of clinical expression of an *RP2* genotype. |
| Hardcastle et al., 1999 | Mutation-screening | UK | 59 XLRP families / 6 *RP2* variants | M | Mainly truncating variants except p.Arg118His. First description of recurrent p.Arg120Ter variant. | *RP2* variants identified in 10% of XLRP families. |
| Mears et al., 1999 | Mutation-screening | North America | 51 XLRP families / 5 *RP2* variants | M | Five truncating variants identified, four novel. | Confirmed truncating variants as an important class of *RP2* variants. |
| Rosenberg et al., 1999 | Genotype–phenotype study | Denmark | 3 *RP2* families | M/F | p.Gln26Ter, p.Arg118His, and in-frame ΔSer6. | Genotype–phenotype differences reported, including a milder phenotype associated with ΔSer6. |
| Sharon et al., 2000 | Mutation-screening / genotype–phenotype study | USA / Canada | 85 XLRP patients / 9 with *RP2* variants | M | 7 *RP2* variants in 9 patients, thereof 5 judged pathogenic, 1 uncertain and one polymorphism. | Visual acuity, visual-field and ERG comparisons of *RP2*- and *RPGR*-associated disease |
| Miano et al., 2001 | Mutation-screening | Italy / Spain | 50 XLRP families / 5 with pathogenic *RP2* variants | M | Five novel *RP2* variants including missense, splice-site and frameshift variants | Expanded the *RP2* mutation spectrum |
| Sharon et al., 2003 | Mutation-screening / genotype–phenotype study | USA / Canada | 187 unrelated males / 11 with *RP2* variants | M | 10 (likely) pathogenic *RP2* variants*,* 1 polymorphism. | Extended clinical phenotyping of *RP2*- and *RPGR*-associated XLRP. Includes 85 individuals from Sharon et al. 2000. |
| Breuer et al., 2002 | Mutation-screening | North America | 234 XLRP families / 9 *RP2* variants in 10 patients | M/F | Two frameshift, one stop, three missense, one splice site and one indel variant. | Extended the *RP2* and *RPGR* variant spectrum. Includes patients from Mears et al. 1999. |
| Dandekar 2004 | Family-based study | UK | 1 family |  | *RP2* c.796–799delCAGA p. (Gln266fsTer269) | Atypical phenotype of macular and peripapillary retinal atrophy. |
| Pelletier et al., 2007 | Mutation-screening / genotype–phenotype study | France | 127 XLRP families/14 *RP2* variants | M/F | 14 *RP2* variants, 12 novel. including frameshift, stop, missense, and splice-site variants | Expanded the *RP2* mutation spectrum |
| Jayasundera et al., 2010 | Genotype–phenotype study | USA | 611 XLRP individuals / 18 *RP2* patients + 7 published cases | M/F | Broad mutational spectrum including frameshift, stop, missense, and splice-site variants | Phenotypic characterization of males and females. Recommendation to screen *RP2* in young affected males with low BCVA, high myopia and early macular involvement. Includes patients from Mears et al. 1999 and Breuer et al. 2002. |
| Jiang et al., 2017 | Family-based study | China | 5 XLRP families / 1 RP2 deletion. | M/F | 17Kb deletion including exon 4 and exon 5 of *RP2* | Novel large deletion removing two exons of *RP2*. |
| Kurata et al., 2019 | Genotype–phenotype study | Japan | 12 XLRP families / 5 with *RP2* variants | M/F | Five *RP2* variants / 2 novel | Clinical and genetic characterization of affected males and female carriers. |
| Fujinami et al., 2020 | Genotype–phenotype study | Japan | Four male individuals with *RP2* variants + literature review. | M/F | Four *RP2* variants, including 2 novel missense variants. | Proposed genotype–phenotype correlations: worse VA loss in patients with null variants based on 10 patients with BCVA data. |
| Cheloni et al., 2022 | Natural-history study | UK | 47 affected males | M | Broad spectrum of pathogenic *RP2* variants | Natural history and longitudinal clinical follow up. |
| Georgiou et al., 2023 | Natural-history study | UK / USA | 54 affected males | M | Multiple pathogenic *RP2* variants | Detailed natural history and structural/functional retinal phenotype. |
| Kuruvilla et al., 2023 | Genotype–phenotype study | New Zealand | 32 XLRP families / 6 *RP2* families | M/F | Four previously unreported *RP2* variants among the *RP2* families | Clinical and genetic characterization of *RP2*- and RPGR-associated disease, including female manifestations |
| Lee et al. 2023 | Case report | Korea | 1 patient | F | c.803dup p.(Glu269Glyfs*7) | Asymmetric presentation in an affected female. |
| Saeed et al., 2023 | Meta-analysis | NA | 35 carrier females + 28 affected males from 13 studies | M/F | Published *RP2* genotypes | Cross-sectional comparative meta-analysis between carrier females and affected males regarding VA and genotype-phenotype. No significant genotype-phenotype correlations were identified |
| Georgiou et al., 2024 | Natural-history study | UK / USA | 27 females from 21 pedigrees. | F | Previously reported pathogenic *RP2* variants | Detailed clinical phenotype and natural history of female carriers. |
| Choi et al. 2024 | Family-based study | Korea | 1 family | M | *RP2* exon 1 deletion | Complex familial cases with concomitant *TGFBI* variant |
| Sun et al. 2026 | Family-based study | China | 23 individuals from one pedigree (6 affected males, 5 carriers, 12 unaffected) | M/F | c.392G>A (p.C131Y) | 8-years follow-up including BCVA, refraction, OCT and ERG. |

**Abbreviations:** BCVA, best corrected visual acuity; ERG, electroretinography; F, female; M, male; NA, not applicable OCT, optical coherence tomography UK, United Kingdom; USA, United States of America; VA, visual acuity; XLRP, X-linked retinitis pigmentosa.
